# Aggregation-prone c9FTD/ALS poly(GA) RAN-translated proteins cause neurotoxicity by inducing ER stress

**DOI:** 10.1007/s00401-014-1336-5

**Published:** 2014-08-31

**Authors:** Yong-Jie Zhang, Karen Jansen-West, Ya-Fei Xu, Tania F. Gendron, Kevin F. Bieniek, Wen-Lang Lin, Hiroki Sasaguri, Thomas Caulfield, Jaime Hubbard, Lillian Daughrity, Jeannie Chew, Veronique V. Belzil, Mercedes Prudencio, Jeannette N. Stankowski, Monica Castanedes-Casey, Ena Whitelaw, Peter E. A. Ash, Michael DeTure, Rosa Rademakers, Kevin B. Boylan, Dennis W. Dickson, Leonard Petrucelli

**Affiliations:** 1 Department of Neuroscience, Mayo Clinic Florida, Jacksonville, FL 32224 USA; 2 Mayo Graduate School, Mayo Clinic College of Medicine, Rochester, MN 55905 USA; 3 Department of Pharmacology, Boston University School of Medicine, Boston, MA 02118 USA; 4 Department of Neurology, Mayo Clinic Florida, Jacksonville, FL 32224 USA

**Keywords:** Amyotrophic lateral sclerosis, *C9ORF72*, Expanded repeat, Frontotemporal dementia, Repeat-associated non-ATG translation, Poly(GA) proteins, Proteasome activity, ER stress

## Abstract

The occurrence of repeat-associated non-ATG (RAN) translation, an atypical form of translation of expanded repeats that results in the synthesis of homopolymeric expansion proteins, is becoming more widely appreciated among microsatellite expansion disorders. Such disorders include amyotrophic lateral sclerosis and frontotemporal dementia caused by a hexanucleotide repeat expansion in the *C9ORF72* gene (c9FTD/ALS). We and others have recently shown that this bidirectionally transcribed repeat is RAN translated, and the “c9RAN proteins” thusly produced form neuronal inclusions throughout the central nervous system of c9FTD/ALS patients. Nonetheless, the potential contribution of c9RAN proteins to disease pathogenesis remains poorly understood. In the present study, we demonstrate that poly(GA) c9RAN proteins are neurotoxic and may be implicated in the neurodegenerative processes of c9FTD/ALS. Specifically, we show that expression of poly(GA) proteins in cultured cells and primary neurons leads to the formation of soluble and insoluble high molecular weight species, as well as inclusions composed of filaments similar to those observed in c9FTD/ALS brain tissues. The expression of poly(GA) proteins is accompanied by caspase-3 activation, impaired neurite outgrowth, inhibition of proteasome activity, and evidence of endoplasmic reticulum (ER) stress. Of importance, ER stress inhibitors, salubrinal and TUDCA, provide protection against poly(GA)-induced toxicity. Taken together, our data provide compelling evidence towards establishing RAN translation as a pathogenic mechanism of c9FTD/ALS, and suggest that targeting the ER using small molecules may be a promising therapeutic approach for these devastating diseases.

**Electronic supplementary material:**

The online version of this article (doi:10.1007/s00401-014-1336-5) contains supplementary material, which is available to authorized users.

## Introduction

Frontotemporal dementia (FTD) and amyotrophic lateral sclerosis (ALS) are devastating neurodegenerative disorders. Despite the fact that FTD presents with changes in behavior, personality and language and ALS is a motor neuron disease which leads to progressive paralysis, there is genetic, neuropathological and clinical overlap between them. Not only do FTD and ALS frequently occur in the same family, many ALS patients develop FTD-like cognitive and behavioral impairments [22, 39, 54], and as many as half of FTD patients develop motor neuron dysfunction [39]. Neuropathologically, neuronal and glial inclusions of TDP-43 are found in most ALS cases, as well as in the most common pathological subtype of FTD [frontotemporal lobar degeneration with TDP-43 pathology (FTLD-TDP)]. Because of this overlap, ALS and FTLD-TDP are considered part of a disease spectrum. This concept was recently reinforced with the discovery that a G_4_C_2_·G_2_C_4_ repeat expansion in a non-coding region of the *C9ORF72* gene is the most common genetic cause of ALS and FTLD-TDP [14, 41, 58].

How the repeat expansion in *C9ORF72* causes “c9FTD/ALS” is not yet definitively known, but many advances have been made since the discovery of this mutation in 2011 (see [21] for review). Potential mechanisms include loss of C9ORF72 function due to epigenetic changes resulting in decreased *C9ORF72* mRNA expression [5, 73]. In addition, repeat-containing RNAs bidirectionally transcribed from the expanded repeat are thought to contribute to disease pathogenesis. The binding of these transcripts by various RNA-binding proteins (RBPs) may impair the ability of these RBPs to interact with their respective RNA targets. Because the repeat-containing transcripts form nuclear RNA foci, RBPs that bind these transcripts may be sequestered therein, also resulting in their loss of function. Furthermore, we and others have shown that transcripts of expanded G_4_C_2_ and G_2_C_4_ repeats undergo repeat-associated non-ATG (RAN) translation [3, 20, 43, 44, 78], an unconventional mode of translation that occurs in the absence of an initiating ATG and in all possible reading frames, first described by Ranum and colleagues [77]. RAN translation of expanded G_4_C_2_ and G_2_C_4_ repeats leads to the synthesis of 6 “c9RAN proteins” of repeating dipeptides: poly(GA) and poly(GR) from sense G_4_C_2_ repeats, poly(PR) and poly(PA) from antisense G_2_C_4_ repeats, and poly(GP) proteins from both sense and antisense transcripts.

Neuronal inclusions of c9RAN proteins are now considered a hallmark of c9FTD/ALS. While this implicates RAN translation as a mechanism of disease, confirmatory data are lacking. The Ranum group has shown that poly(PR) and poly(GP) proteins induce cellular toxicity in cultured cells independently of the accumulation of RNA foci [78], suggesting that c9RAN protein expression may indeed be detrimental. However, given that inclusions of poly(GP) proteins are present in some, but not all, affected regions of the central nervous system (CNS) in c9FTD/ALS [3, 20], and a recent study showing that poly(GA) pathology, unlike TDP-43 pathology, does not correlate with the degree of neurodegeneration in c9FTD/ALS [40], put into question the contribution of c9RAN proteins to disease pathogenesis. Conversely, the discovery of a c9FTD kindred with early intellectual disability and extensive poly(GA) inclusions but little, if any, TDP-43 pathology [56], provides compelling evidence that c9RAN proteins, or at least poly(GA) proteins, are harmful. Like poly(GP) inclusions, inclusions of poly(GA) appear to be abundant in c9FTD/ALS [37, 40, 43, 44, 56], perhaps because of the hydrophobic nature of the protein. Using various models, the present study thus sought to evaluate the neurotoxic potential of poly(GA) protein expression and aggregation, as well as the mechanism(s) driving this toxicity.

## Materials and methods

### Generation of plasmids

To generate expression vectors for GFP-(GA)_50_, GFP-(GP)_47_, GFP-(GR)_50_, GFP-(PR)_50_ or GFP-(PA)_50_, gene fragments containing individual dipeptide repeats (Table 1) were synthesized by GeneArt and ligated to the HindIII and BamHI restriction sites of a pEGFP-C1 vector (Clontech Laboratories) in frame with the EGFP coding sequence. To generate the AAV1-GFP-(GA)_50_ expression vector, the EGFP coding sequence with restriction sites identical to those in pEGFP-C1 and containing a stop codon in each frame downstream of the multiple cloning site was cloned into the AAV expression vector pAM/CBA-pl-WPRE-BGH (“pAAV”). Gene fragments from the gene synthesis were cloned into the HindIII and BamHI restriction sites of the pAAV EGFP fusion vector in frame with the EGFP coding sequence. To generate the GST-(GA)_50_ vector, PCR was performed to synthesize cDNA of a (GA)_50_ fragment, which was then inserted into a pGEX-6P-1 vector (GE Healthcare) using BamHI and XhoI cloning sites. To generate the ATG-(GA)_50_-V5 vector, cDNA of the (GA)_50_ fragment with an ATG start codon was inserted into a pcDNA6-V5-His vector (Life Technologies) using HindIII and XhoI cloning sites. To generate the GFP-c9(GA)_50_ expression vector, a gene fragment containing 50 pathological G_4_C_2_ repeats was produced as previously described [33], and then ligated to EcoRV restriction sites of a pcDNA6-V5-His vector (Life Technologies). The GFP sequence was inserted upstream of (G_4_C_2_)_50_ using HindIII and EcoRI cloning sites to drive protein expression in the GA reading frame. To generate pAG3-6 × stops-(±)ATG-(GA)_50_-3T vectors, cDNA of the (GA)_50_ fragment with or without an ATG start codon was inserted in a pAG3-6 × stops-3T vector between the 6 stop codons and the 3 tags (Online Resource 4a). The sequence of all plasmids was verified by sequence analysis.

**Table 1:**
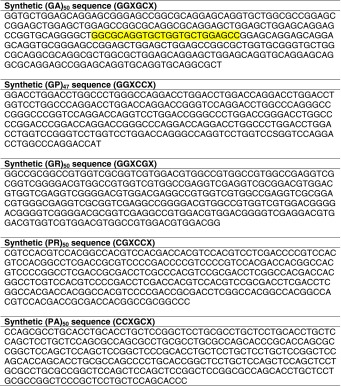
Synthetic cDNA sequences used for expression vectors

The target sequence of the FISH probe for the synthetic (GA)_50_ sequence is highlighted in yellow

### Purification of recombinant proteins and generation of anti-poly(GA) antibody

GST or GST-(GA)_50_ plasmids were used for transformation in Rosetta™(DE3)pLysS competent cells (EMD4Biosciences). To induce expression of recombinant proteins, bacteria were cultured overnight at 16 °C in the presence of 0.5 mM isopropyl *β*-d-1-thiogalactopyranoside (IPTG). After centrifugation, the bacteria pellet was washed with phosphate-buffered saline (PBS), and then lysed on ice for 30 min with PBS containing 1 % Triton X-100 (PBST). After sonication, the lysates were centrifuged at 18,000*g* for 30 min. The resulting supernatant was applied to a Glutathione Sepharose 4B column (GE Healthcare). After washing the column with PBST, the recombinant proteins were eluted from the column using Tris–HCl (50 mM, pH 8.0) containing 20 mM reduced glutathione. Afterwards, the solution was dialyzed with 50 mM Tris–HCl (50 mM, pH 8.0) to remove glutathione, and then concentrated. Recombinant GST-(GA)_50_ protein was used as the antigen to produce rabbit polyclonal antibodies. Pre-immune serum from rabbits was tested against brain tissue from c9FTD/ALS cases by immunohistochemistry and confirmed negative. Antiserum was used directly for Western blot and immunohistochemistry studies.

### Immunohistochemistry and semi-quantitative analysis of poly(GA) inclusions in c9FTD/ALS

Neuropathological assessment was performed on 10 cases with the expanded *C9ORF72* hexanucleotide repeat. These cases were neuropathologically diagnosed as frontotemporal lobar degeneration (FTLD; *N* = 4), amyotrophic lateral sclerosis (ALS; *N* = 4), or both (FTLD–MND; *N* = 2). Four regions of formalin-fixed paraffin-embedded tissue from the left hemi-brain were selected: the midfrontal gyrus of the frontal lobe, the posterior hippocampus at the level of the lateral geniculate nucleus (including occipitotemporal gyrus), the thalamus at the level of the subthalamic nucleus, and the cerebellum with the dentate nucleus. Tissue was cut into 6-µm-thick sections, mounted on glass slides, and dried overnight. Slides were subsequently deparaffinized and immunohistochemistry was performed using 30 min of antigen retrieval (steam), Dako EnVision+ reagents and Autostainer (DAKO), and the anti-poly(GA) rabbit polyclonal antibody (1:50,000). Following immunohistochemistry, all slides were counterstained with Lerner’s hematoxylin, dehydrated, and coverslipped. Slides were analyzed semi-quantitatively (Table 2) on an Olympus BX40 microscope (Olympus America). Neuropathologic burden was graded on a 4-point scale: sparse (±), mild (+), moderate (++), and severe (+++). In densely neuronal-populated regions, such as the internal granule cell layer of the cerebellum and the dentate fascia of the hippocampus, the pathologic grade was validated using averaged counts in 40× and 20× magnification microscopic fields, respectively. Slides were imaged using a Zeiss AxioImager Z1 microscope (Carl Zeiss Microscopy).

**Table 2:** Poly(GA) neuropathology in c9FTD/ALS cases

Path Dx	Case #	Hippocampus		Temporal cortex	Frontal cortex	Thalamus	Cerebellum	
		DF	CA3/2				ML	GCL
FTLD	1	++	++	++	++	++	++	+++
FTLD	2	+	+	++	++	±	+	++
FTLD	3	+	+	+	+	++	++	+
FTLD	4	++	++	++	+	++	+	+++
FTLD–MND	5	++	++	+++	+++	+	++	+++
FTLD–MND	6	+	+	+	++	++	±	++
ALS	7	++	+	++	++	++	+	**
ALS	8	+++	+	++	++	+	++	+++
ALS	9	++	++	++	++	±	++	+++
ALS	10	++	++	++	++	+	++	++

± Sparse, + mild, ++ moderate, +++ severe, ** cells autolyzed, *CA3/2* hippocampus proper, *DF* dentate fascia, *GCL* granule cell layer, *ML* molecular layer, *Path Dx* neuropathologic diagnosis

### Electron microscopy (EM) and immuno-electron microscopy (immuno-EM)

To examine the filamentous structure of recombinant GST-(GA)_50_ proteins, recombinant GST or GST-(GA)_50_ proteins were diluted to 1 µg/µl in 20 mM Tris–HCl, pH 7.5, 50 mM KCl, 10 mM MgCl_2_ in a final volume of 30 µl. The samples were incubated at 30 °C for 6 h, and then diluted to 0.1 µg/µl by reaction buffer and loaded onto grids for regular EM analysis. For immuno-EM analysis, mouse monoclonal anti-GST antibody (1:20, Thermo Scientific) was used as primary antibody, and goat anti-mouse IgG conjugated with 6 nm colloidal gold particles (1:20, Jackson ImmunoResearch Laboratories) was used as the secondary antibody. To examine the filamentous structure of poly(GA) proteins in c9FTD/ALS patients, small pieces (1.5 × 1.5 × 1 mm) of cerebellar folia or hippocampus from formalin-fixed brains were dissected and processed for routine electron microscopy (EM) or post-embedding immunogold EM as previously described [34]. Rabbit polyclonal anti-poly(GA) antibody (1:50) was used as a primary antibody and goat anti-rabbit IgG conjugated with 18 nm colloidal gold particles (1:20, Jackson ImmunoResearch Laboratories) was used as the secondary antibody. Thin sections stained with uranyl acetate and lead citrate were examined with a Philips 208S electron microscope (FEI) fitted with a Gatan 831 Orius CCD camera (Gatan). Digital images were processed with Adobe Photoshop CS5 software.

### Preparation of urea fractions and dot blot analysis of frozen cerebellar tissue

The urea fraction of human tissues was prepared as previously described [46, 75]. In brief, ~100 mg frozen postmortem cerebellar tissue from carriers and non-carriers of the *C9ORF72* repeat expansion was subjected to a sequential extraction protocol using low-salt buffer, high salt–Triton X-100 buffer, myelin floatation buffer, and sarkosyl buffer. Sarkosyl-insoluble material was finally extracted in urea buffer and saved as the urea fraction. For dot blots, urea fractions (2 µl per sample) were dotted directly onto nitrocellulose membrane. After incubation at 37 °C for 30 min, the membrane was blocked with 5 % nonfat dry milk in Tris-buffered saline containing 0.1 % Triton X-100 (TBST) for 1 h, then incubated with rabbit polyclonal anti-poly(GA) antibody (1:1000) overnight at 4 °C. The membrane was washed in TBST, and then incubated with donkey anti-rabbit IgG conjugated to horseradish peroxidase (1:5000, Jackson ImmunoResearch Laboratories) for 1 h. Protein expression was visualized by enhanced chemiluminescence treatment and exposure to a film.

### Preparation of cell lysates

HEK293T cells grown in 6-well plates were transfected for 48 h with 1 µg of an expression vector [GFP or GFP-(GA)_50_] or 5 µg of an expression vector [ATG-(GA)_50_-V5, ATG-(GA)_100_-V5, ATG-(GA)_50_-3T or (GA)_50_-3T] using Lipofectamine 2000 (Life Technologies). To prepare Triton X-100 soluble and insoluble fractions, cell pellets expressing GFP or GFP-(GA)_50_ were lysed in Co-IP buffer (50 mM Tris–HCl, pH 7.4, 300 mM NaCl, 1 % Triton X-100, 5 mM EDTA) containing PMSF as well as protease and phosphatase inhibitors. Lysates were sonicated on ice, and then centrifuged at 16,000*g* for 20 min. Supernatants were saved as Triton X-100-soluble fractions. The insoluble pellets were dissolved in Co-IP buffer plus 2 % SDS, PMSF, and both a protease and phosphatase inhibitor mixture. After sonication, lysates were centrifuged at 16,000*g* for 20 min, and these supernatants were saved as Triton X-100-insoluble fractions. To prepare total cell lysates, pellets from cells transfected with ATG-(GA)_50_-V5, ATG-(GA)_100_-V5, ATG-(GA)_50_-3T or (GA)_50_-3T constructs were lysed in Co-IP buffer plus 2 % SDS, PMSF, and both protease and phosphatase inhibitor mixture. After sonication, lysates were centrifuged at 16,000*g* for 20 min. The protein concentration of supernatants was determined by BCA assay (Thermo Scientific) prior to Western blot analysis.

### Fluorescence in situ hybridization (FISH)

To examine RNA foci, cells were subjected to FISH. In brief, HEK293T cells grown on glass coverslips in 24-well plates were transfected with 0.5 µg GFP-(GA)_50_ or GFP-c9(GA)_50_ expression vectors made, respectively, with a synthetic sequence [(GGXGCX)_50_; GFP-(GA)_50_] or a pathological sequence [(GGGGCC)_50_; GFP-c9(GA)_50_]. After 24 h, cells were fixed in 4 % paraformaldehyde for 20 min, permeabilized in DEPC-treated PBS-0.2 % Triton X-100 for 10 min, and washed 3 times with DEPC-treated PBS. Cells were hybridized with denatured Cy3-conjugated probe (5′-Cy3/GGCUCCAGCACCAGCACCUGCGCC-3′, 2 ng/µl) for the synthetic sequence, or (5′-Cy3/GGCCCCGGCCCCGGCCCCGGCCCC-3′, 2 ng/µl) for the pathological sequence, overnight at 37 °C. The composition of the hybridization buffer was 50 % formamide, 10 % dextran sulfate, 0.1 mg/ml yeast tRNA, 2 × SSC, 50 mM sodium phosphate. Cells were then washed once with 50 % formamide/1 × SSC for 30 min at 37°C, and twice with DEPC-PBS at room temperature for 5 min. Then, the cells were counterstained with Hoechst 33258 (1 µg/ml, Life Technologies). Immunostained cells were visualized using a Zeiss LSM 510 META confocal microscope.

### Cell culture, immunofluorescence staining and quantification of activated caspase-3-positive cells

HEK293T cells grown on glass coverslips in 24-well plates were transfected with 0.5 µg of an expression vector [GFP, GFP-(GA)_5_, GFP-(GA)_50_, ATG-(GA)_50_-V5, ATG-(GA)_100_-V5, ATG-(GA)_50_-3T or (GA)_50_-3T] using Lipofectamine 2000 (Life Technologies). After 48 h, media was collected for analysis using an LDH assay (Promega) to assess cell toxicity. In addition, cells were fixed with 4 % paraformaldehyde in PBS for 15 min, and then permeabilized with PBS–0.5 % Triton X-100 for 10 min. To examine caspase-3 activation, cells were blocked with 5 % nonfat milk for 1 h at room temperature, then incubated overnight at 4 °C with rabbit polyclonal anti-activated caspase-3 antibody (9661, 1:250, Cell signaling) and mouse monoclonal anti-HA antibody (clone 3F10, 1:1000, Roche; used for cells expressing HA-tagged proteins) or mouse monoclonal anti-V5 antibody (R960-25, 1:2000, Life Technologies; used for cells expressing V5-tagged proteins). To determine whether poly(GA) inclusions are ubiquitin- and p62-positive, cells expressing ATG-(GA)_100_-V5 were incubated with rabbit polyclonal anti-V5 antibody (V8137, 1:2000, Sigma) and mouse monoclonal anti-ubiquitin (clone Ubi-1, 1:100, EMD Millipore) or mouse monoclonal anti-p62 (610833, 1:100, BD Biosciences) antibodies. To examine the ER-Golgi distribution of GFP-(GA)_50_ inclusions, cells were subjected to immunofluorescence staining using rabbit polyclonal anti-giantin antibody (ab24586, 1:250, Abcam) or mouse monoclonal anti-KDEL antibody (10C3, 1:500, StressGen). After washing, cells were incubated with the corresponding Alexa Fluor 488 donkey anti-mouse and Alexa 568-conjugated donkey anti-rabbit secondary antibody (1:500, Molecular Probes) at room temperature for 2 h. Hoechst 33258 (1 µg/ml, Life Technologies) was used to stain cellular nuclei. Images were obtained on a Zeiss LSM 510 META confocal microscope. To quantify the number of cells positive for activated caspase-3 staining, coverslips mounted on slides were scanned by Aperio ScanScope. Sixteen fields were randomly selected under 20× magnification. For each field, the number of activated caspase-3-positive cells and the number of GFP-positive cells were counted in a blinded fashion using MetaMorph software. These counts were used to calculate the average percentage of activated caspase-3-positive cells in cells expressing GFP, GFP-(GA)_5_ or GFP-(GA)_50_.

### Primary neuronal culture preparation, quantification of neurite outgrowth and immunofluorescence staining

Primary neuronal cultures were prepared as previously described [76]. In brief, the cortex from embryonic day 18 (E18) mice was dissected and digested. Following centrifugation to collect the cell pellet, cells were resuspended in Neurobasal A (Life Technologies) supplemented with B27, GMAX, gentamicin and bFGF (Life Technologies). Neurons were seeded at a density of 9 × 10^3^ cells/well in 96-well plates, 2 × 10^4^ cells/coverslip in 24-well plates, or 7 × 10^5^ cells/well in 6-well plates. At day in vitro 4, neurons were transduced to express GFP, GFP-(GA)_50_, or ATG-(GA)_100_-V5. The following numbers of rAAV1 genome particles were used for 96-, 24- and 6-well plates, respectively: 1 × 10^9^, 1.2 × 10^9^, and 1 × 10^10^. Quantification of neurite outgrowth was performed as previously described [19, 76], using neurons in 96-well plates immunostained 5 days post-transduction with mouse monoclonal anti-MAP2 antibody (M9942, 1:1000, Sigma) and rabbit polyclonal anti-V5 antibody (V8137, 1:2000, Sigma) (used for cells expressing V5-tagged proteins). The average total neurite length per neuron was calculated for all groups using counts obtained in a blinded fashion. Caspase-3 activation was evaluated using neurons on glass coverslips in 24-well plates. Seven days post-transduction, neurons were fixed and immunostained using a rabbit polyclonal anti-activated caspase-3 antibody (9661, 1:250, Cell signaling) and/or mouse monoclonal anti-V5 antibody (R960-25, 1:2000, Life Technologies). Western blot analysis and LDH studies were conducted using neurons grown in 6-well plates. Seven days post-transduction, media was collected for the LDH assay, and cells were lysed in Co-IP buffer with 2 % SDS, PMSF, as well as protease and phosphatase inhibitors. Lysates were sonicated and centrifuged at 16,000*g* for 20 min. Supernatants were saved and the protein concentration was determined by BCA assay. Samples were analyzed by Western blot. Some cultures were exposed to different treatments. Non-transduced neurons were treated with tunicamycin (10 µg/µl, Sigma) or MG-132 (10 µM, EMD4Biosciences) at day in vitro 6, then harvested the next day. Neurons expressing GFP-(GA)_50_ were treated with a fresh preparation of TUDCA (0.25 or 0.5 mM, EMD4Biosciences) 1 day after transduction or salubrinal (2.5 or 5.0 µM, Sigma) 3 days after transduction. Seven days after transduction, TUDCA- or salubrinal-treated neurons were harvested for analysis.

### Proteasome activity assay

Proteasome activity assays were performed as previously described [61]. In brief, pellets of neurons subjected to different treatments were lysed in 350 μl of assay buffer (10 mM Tris–HCl, 0.5 mM DDT, 5 mM ATP, 0.035 % SDS, 5 mM MgCl_2_, pH 7.8). After homogenization, cell lysates were centrifuged at 1,000*g* for 10 min. The supernatants were collected and the protein concentration was determined by Bradford assay (Thermo Scientific). Then, the proteasome substrate Suc-Leu-Leu-Val-Tyr-AMC was added to 300 μl of lysate at a final concentration of 40 μM. The reaction was started by incubating samples at 37°C for 30 min, and then terminated by adding 75 μl H_2_O and 11.25 μl ethanol to quench the substrate. After centrifugation at 1,000*g* for 3 min, 100 μl of each sample was added into a black 96-well plate in triplicate. Fluorescence was measured at 360 nm excitation and 465 nm emission. Data was normalized to the GFP control group.

### Live cell imaging

HEK293T cells grown in 96-well plates were transfected with 0.1 µg of GFP-(GA)_50_ vector using Lipofectamine 2000 (Life Technologies). To monitor inclusion formation 12 h post-transduction, images of live cells were obtained every 5 min for 5 h using the BD pathway 855. The resulting images were combined to create the video shown in the Online Resources section.

### Western blot analysis

Western blot analysis was performed as previously described [75, 76]. In brief, samples were heated in Laemmli’s buffer, and equal amounts of protein were loaded into 10-well 10 % or 4–20 % Tris–glycine gels (Novex). After transfer, blots were blocked with 5 % nonfat dry milk in TBST for 1 h, then incubated with a rabbit polyclonal anti-GFP antibody (A-6455, 1:2000, Life Technologies), rabbit polyclonal anti-activated caspase-3 antibody (9661, 1:1000, Cell signaling), mouse monoclonal anti-V5 antibody (R960-25, 1:2000, Life Technologies), rabbit polyclonal anti-poly(GA) serum (1:4000), mouse monoclonal anti-GST antibody (MA4-004, 1:2000, Thermo Scientific), mouse monoclonal anti-HA antibody (clone 3F10, 1:1000, Roche), mouse monoclonal anti-Myc antibody (clone 9E10, 1:1000, Roche), mouse monoclonal anti-Flag antibody (F3165, 1:1000, Sigma), rabbit polyclonal anti-ubiquitin antibody (Z0458, 1:1000, Dako), mouse monoclonal anti-BIP antibody (610978, 1:1000, BD Biosciences), rabbit polyclonal anti-CHOP antibody (sc-575, 1:200, Santa Cruz Biotechnology), rabbit polyclonal anti-phospho-PERK (sc-32577, 1:500, Santa Cruz Biotechnology), rabbit polyclonal anti-PERK (sc-13073, 1:1000, Santa Cruz Biotechnology), rabbit polyclonal anti-ATF6 (sc-22799, 1:1000, Santa Cruz Biotechnology), rabbit polyclonal anti-phospho-eIF2α (9721, 1:500, Cell signaling) or mouse monoclonal GAPDH antibody (H86504 M, 1:10000, Meridian Life Science) overnight at 4°C. Membranes were washed in TBST and incubated with donkey anti-rabbit or anti-mouse IgG conjugated to horseradish peroxidase (1:5000; Jackson ImmunoResearch) for 1 h. Protein expression was visualized by enhanced chemiluminescence treatment and exposure to film.

### RNA extraction, semi-quantitative PCR and quantitative PCR (qRT-PCR)

Total RNA was extracted from HEK293T cells, neurons or frozen postmortem frontal cortex tissues using the RNAeasy Plus Mini Kit (QIAGEN) as per the manufacturer’s instructions, combined with an in-column DNase I digestion step. RNA integrity was obtained using the Agilent 2100 Bioanalyzer (Agilent Technologies). cDNA was obtained after reverse-transcription polymerase chain reactions using 500–2000 ng of RNA with random primers and the High Capacity cDNA Transcription Kit (Applied Biosystems) as per the manufacturer’s instructions. To examine XBP1 splicing in neurons, 2 μl of cDNA was used in a 20 μl reaction according to the manufacturer’s protocol for a Taq PCR Core kit (Qiagen). The PCR primers for XBP1 and GAPDH were used as in previously described [28]. The amplification conditions consisted of an initial denaturation step at 95 °C for 5 min; 10 cycles of 94 °C for 20 s, 65–55 °C touchdown for 20 s, and 72 °C for 30 s; 35 cycles of 94 °C for 20 s, 58 °C for 20 s, and 72 °C for 30 s, and a final extension at 72 °C for 5 min. The PCR products were run on 2 % agarose gels for 35 min at 135 V. The intensity of unspliced and spliced XBP1 bands was quantified by densitometric analysis using Fujifilm MultiGauge software, and then the percentage of unspliced or spliced XBP1 was calculated.

To quantify mRNA levels of GFP, GFP-(GA)_5_ and GFP-(GA)_50_ in HEK293T cells or neurons, quantitative PCR (qRT-PCR) was conducted in triplicate for all samples using SYBR green assay (Life Technologies) on an ABI Prism 7900HT Fast Real-Time PCR System (Applied Biosystems). The primers used were: GFP: 5′-GAAGCGCGATCACATGGT-3′ and 5′-CCATGCCGAGAGTGATCC-3′; GAPDH: 5′-CATGGCCTTCCGTGTTCCTA-3′ and 5′-CCTGCTTCACCACCTTCTTGAT-3′. The mRNA values of GFP, GFP-(GA)_5_ and GFP-(GA)_50_ were normalized to GAPDH values, an endogenous transcript control. To quantify ER stress markers in frontal cortex tissues from ALS patients with or without the *C9ORF72* repeat expansion (Table 3), qRT-PCR was performed using TaqMan assays (Life Technologies) for ATF4 (Hs00909569_g1), CHOP (Hs00358796_g1), or BIP (Hs00946350_g1), or a SYBR green assay for RPLP0 (primers: 5′-TCTACAACCCTGAAGTGCTTGAT-3′ and 5′-CAATCTGCAGACAGACACTGG-3′). The mRNA values of ATF4, CHOP, and BIP were normalized to RPLP0 values, an endogenous transcript control. Since it has been reported that GAPDH expression is altered in neurodegenerative diseases [11], we evaluated several endogenous controls by qRT-PCR in the frontal cortex regions and RPLP0 was chosen because this transcript presented overall low C_t_ values and lowest C_t_ variation (unpublished data). In addition, RPLP0 has been used as a stable reference gene in previous studies [1, 16, 31].

**Table 3:** ALS cases with or without the *C9ORF72* repeat expansion used for qPCR analysis of ER stress markers

Path Dx	Case # (continued from Table 2)	*C9ORF72* repeat expansion	Gender	Age of onset	Age of death
c9ALS	7	Y	M	52	58
c9ALS	9	Y	F	n/a	49
c9ALS	10	Y	F	n/a	41
c9ALS	11	Y	F	61	64
c9ALS	12	Y	M	56	58
c9ALS	13	Y	M	49	53
c9ALS	14	Y	F	n/a	50
c9ALS	15	Y	F	n/a	50
ALS	16	N	M	45	50
ALS	17	N	M	53	58
ALS	18	N	F	n/a	53
ALS	19	N	M	n/a	47
ALS	20	N	F	n/a	60
ALS	21	N	F	n/a	65
ALS	22	N	F	n/a	69
ALS	23	N	F	n/a	70
ALS	24	N	F	n/a	72
ALS	25	N	F	n/a	79
ALS	26	N	F	n/a	80

## Results

### Poly(GA) proteins form abundant inclusions in c9FTD/ALS

**Fig. 1:**
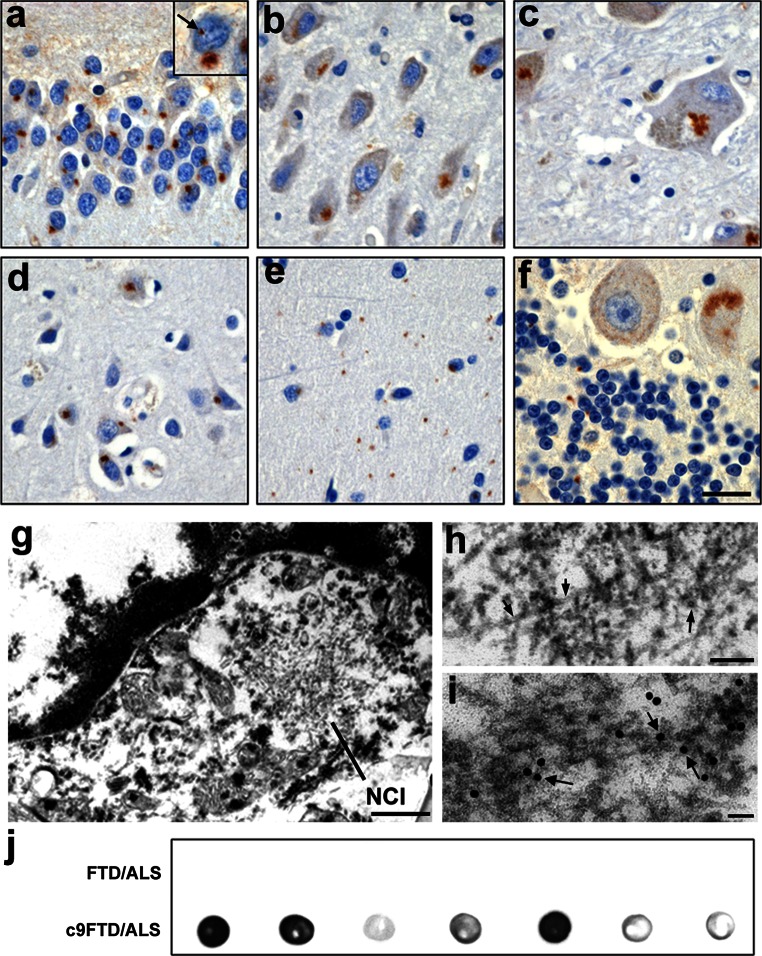
Neuropathology of c9RAN poly(GA) proteins in *C9ORF72* repeat expansion cases. **a**–**f** Immunohistochemical analysis shows that poly(GA) proteins accumulate throughout the central nervous system of *C9ORF72* repeat expansion carriers as neuronal cytoplasmic inclusions and neuronal intranuclear inclusions (*arrow* in insert, **a**). Regions with a particularly high burden include the dentate fascia of the hippocampus (**a**; Case 8), the hippocampus proper (**b**; CA3/2; Case 10), the anterior thalamus (**c**; Case 4), the frontal cortex (**d**; Layers IV–V; Case 7), the cerebellar molecular layer (**e**; Case 1) and the cerebellar internal granule cell layer (**f**; Case 9). *Scale bar* represents 25 µm (**a**–**e**) and 20 µm (**f**). Regular electron microscopy (EM) of granule cells of the cerebellar cortex from a c9FTD-MND case shows that cytoplasmic inclusions (**g**) are composed of 15–17 nm filaments (*arrow*, **h**). Immuno-EM with anti-poly(GA) antibody labeled with gold particles (18 nm) reveals poly(GA) proteins localize to filaments (*arrow*, **i**). *Scale bars* represent 0.5 μm (**g**), 100 nm (**h**), and 50 nm (**i**). **j** Dot blot reveals that anti-poly(GA) immunoreactivity in cerebellar urea fractions is specific to c9FTD/ALS. Each dot represents one case

To investigate the contribution of poly(GA) inclusions to c9FTD/ALS pathogenesis, we generated a rabbit polyclonal antibody using recombinant GST-(GA)_50_ protein as the antigen. Analysis of recombinant GST-(GA)_50_, in comparison to GST alone, confirmed that it forms high molecular weight species (arrow, Online Resource 1a) and filamentous structures in vitro (Online Resource 1b and c), rendering it an ideal antigen for the production of antibodies to detect poly(GA) pathology. Indeed, the resulting antibodies, which specifically detect poly(GA) protein and no other c9RAN proteins (Online Resource 1d), stain inclusions throughout the CNS of c9FTD/ALS cases (Fig. 1a–f; Table 2) but not age-matched FTLD-TDP and ALS cases without the *C9ORF72* repeat expansion (Online Resource 1e). Further confirming specificity of this novel polyclonal poly(GA) antibody, immunostaining c9FTD/ALS tissues with pre-immune serum was negative (Online Resource 1f). The distribution of anti-poly(GA) immunoreactive inclusions in c9FTD/ALS (Table 2) paralleled that of poly(GP) inclusions, which we previously reported to be enriched in the cerebellum, hippocampus, and neocortex [3]. Poly(GA) inclusions were predominantly neuronal cytoplasmic inclusions (NCI), but neuronal intranuclear inclusions (NIIs) (arrow in insert, Fig. 1a) were also observed. Electron microscopy (EM) studies of NCI in granule cells of the cerebellum (Fig. 1g) showed that the inclusions contain 15–17 nm filaments (arrow, Fig. 1h). Immuno-EM with anti-poly(GA) antibody confirmed the localization of poly(GA) proteins to these filaments (arrow, Fig. 1i). Consistent with these findings, insoluble poly(GA) proteins were detected in urea fractions of c9FTD/ALS cerebellar tissue, but not in cerebellar tissue from FTLD and ALS cases with no expanded repeat, as assessed by dot blot (Fig. 1j).

### Poly(GA) proteins form inclusions and are toxic in cultured cells

**Fig. 2:**
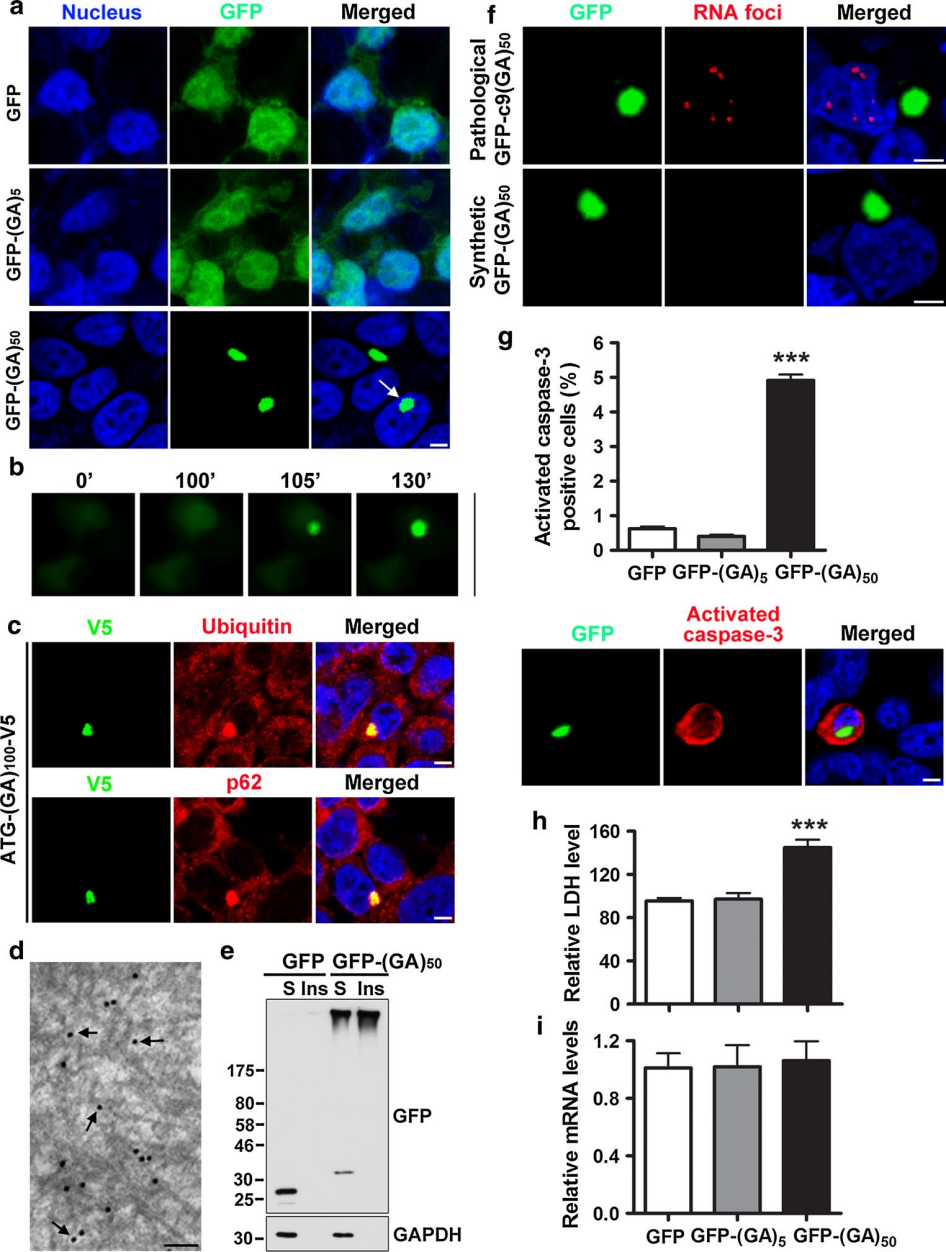
Poly(GA) proteins form inclusions and are toxic in cultured cells. **a** Expression of GFP-(GA)_50_ in cultured HEK293T cells results in the formation of cytoplasmic or nuclear (*arrow*) inclusions. *Scale bar* represents 5 µm. **b** Representative images of live cells demonstrating how quickly inclusions form (compare the image at 100 min to the image at 105 min). **c** Poly(GA) inclusions are ubiquitin and p62-positive in cultured cells. *Scale bar* represents 5 µm. **d** Immuno-electron microscopy with an anti-GFP antibody labeled with gold particles shows that cytoplasmic GFP-(GA)_50_ inclusions are composed of filamentous structures (*arrow*). *Scale bar* represents 100 nm. **e** Western blot analysis of Triton X-100 soluble (S) and insoluble (Ins) cell lysates shows that a portion of poly(GA) proteins forms high molecular weight material. **f** Cultured cells were made to express GFP-(GA)_50_, which encodes a synthetic repeat sequence (GGXGCX)_50_ (where X represents a random nucleotide), or GFP-c9(GA)_50_, in which the pathological repeat sequence (GGGGCC)_50_ was used. Post-transfection, cells were subjected to RNA fluorescence in situ hybridization (FISH) to visualize RNA foci. GFP-(GA)_50_ expression leads to the formation of poly(GA) inclusions, but transcripts from this sequence do not form RNA foci. In contrast, both RNA foci and poly(GA) inclusions are formed in cells that express GFP-c9(GA)_50_. *Scale bar* represents 5 µm. **g** Quantitative analysis and representative image showing that cells bearing inclusions of GFP-(GA)_50_ are immunoreactive for activated caspase-3, a crucial mediator of cell death. *Scale bar* represents 5 µm. **h** LDH activity in media, an indicator of cell toxicity, is increased in cells expressing GFP-(GA)_50_. **i** Transgene mRNA levels are comparable among cells expressing GFP, GFP-(GA)_5_ and GFP-(GA)_50_. Data represent the mean ± SEM from sixteen random selected fields (**g**) or three separate experiments (**h** and **i**). ***P < 0.001, as analyzed by one-way analysis of variance followed by Tukey’s post hoc analysis

In investigating the potential contribution of poly(GA) proteins to neurodegeneration in c9FTD/ALS, we generated expression constructs for CMV-promoted, ATG-initiated translation of synthetic repeats encoding GFP-tagged (GA)_5_ or (GA)_50_. For comparison purposes, we also produced vectors for the expression of all other c9RAN proteins [GFP-(GP)_47_, GFP-(GR)_50_, GFP-(PR)_50_ or GFP-(PA)_50_]. Similar to the intracellular localization of GFP alone, GFP-(GP)_47_ and GFP-(PA)_50_ were diffusely distributed throughout cells, whereas GFP-(GR)_50_ and GFP-(PR)_50_ accumulated into discrete nuclear structures (Online Resource 2). In contrast, GFP-(GA)_50_ formed many inclusions, the majority of which were cytoplasmic, although the occasional nuclear inclusion was observed (arrow, Fig. 2a). These results suggest that, among c9RAN proteins, poly(GA) is especially aggregation prone. The propensity of poly(GA) proteins to aggregate, however, is dependent on repeat length given that GFP-(GA)_5_ remained diffusely distributed in transfected cells (Fig. 2a). Poly(GA) aggregation also appears to be a relatively rapid process. Upon monitoring GFP-(GA)_50_ inclusion formation in living cells, it was observed that, within a span of only 5 min, diffuse GFP-(GA)_50_ can quickly form a small aggregate, which then goes on to become a large inclusion within approximately 30 min (Fig. 2b, Online Resource 3). The aggregation of GFP-(GA)_50_ is not likely the result of a tag artifact since poly(GA) inclusions were also observed in cells expressing ATG-(GA)_100_-V5 (Fig. 2c). Of note, (GA)_100_-V5 inclusions were ubiquitin- and p62-positive (Fig. 2c), reminiscent of poly(GA) pathology in c9FTD/ALS [2, 44]. Likewise, similar to the filaments observed in c9FTD/ALS tissue, GFP-(GA)_50_ inclusions in cultured cells were composed of filamentous structures (arrow, Fig. 2d). Consistent with these findings, Western blot analysis of Triton X-100 soluble and insoluble transfected cell fractions revealed that, while GFP was detected as a monomer in the soluble fraction, GFP-(GA)_50_ accumulated in both soluble and insoluble fractions as high molecular weight species (Fig. 2e), akin to the high molecular weight bands of recombinant GST-(GA)_50_ noted above. Such high molecular weight species were also detected in lysates from cells expressing ATG-(GA)_50_-HA, ATG-(GA)_50_-V5 or ATG-(GA)_100_-V5, again ruling-out the possibility that formation of high molecular weight poly(GA) proteins is driven by the tag to which it is fused (Online Resource 4b and d).

The purpose of generating a vector that encodes a synthetic repeat sequence for the expression of GFP-(GA)_50_, specifically one in which a random nucleotide was introduced in the 3rd and 6th codon positions (GGXGCX), was to enable the evaluation of poly(GA) protein toxicity without the confounding contribution of RNA foci or other c9RAN proteins, the production of which may depend on the secondary structure of G_4_C_2_ repeat-containing RNA [23, 57, 64, 77]. To confirm that the RNA encoded by the synthetic sequence does not result in foci formation, RNA-FISH of GFP-(GA)_50_-expressing cells was undertaken. As above, poly(GA) inclusions were observed in cells expressing GFP-(GA)_50_, but no RNA foci were detected (Fig. 2f). Conversely, both foci and poly(GA) inclusions were found in cells expressing GFP-c9(GA)_50_, which encodes 50 unadulterated G_4_C_2_ repeats (Fig. 2f). Next, to confirm that our synthetic (GA)_50_ sequence does not undergo RAN translation, we generated expression vectors containing the (GA)_50_ sequence with or without an ATG start codon. The vectors include six stop codons upstream of (±)ATG-(GA)_50_ (two in each reading frame) to prevent ATG-initiated translation from the vector sequence, and a different tag in each reading frame downstream of (±)ATG-(GA)_50_ to monitor protein expression in all frames [i.e., frame 1: (GA)_50_-HA; frame 2: novel RAN protein-Myc; frame 3: novel RAN protein-Flag] (Online Resource 4a). Western blot analysis revealed that cells transfected with the ATG-(GA)_50_-3T expression vector produce (GA)_50_-HA proteins but not Myc- or Flag-tagged proteins RAN translated from reading frames 2 or 3 (Online Resource 4b). Moreover, no proteins were expressed from any of the three reading frames in cells transfected with the ATG-free (GA)_50_-3T vector (Online Resource 4b), despite comparable transgene mRNA levels in ATG-(GA)_50_-3T and (GA)_50_-3T expressing cells (Online Resource 4c). Together, our results indicate that our synthetic (GA)_50_ sequence is not RAN translated and results only in the production of poly(GA) proteins.

Having excluded the potential involvement of foci formation and RAN translation in our poly(GA)-expressing cellular model, we next evaluated whether poly(GA) expression and aggregation are associated with cellular toxicity using caspase-3 activation as an indicator of cell death. Compared to the 0.6 % of GFP- or GFP-(GA)_5_-expressing cells that showed signs of activated caspase-3, the percentage of cells positive for activated caspase-3 was approximately eightfold higher upon expression of GFP-(GA)_50_ (Fig. 2g, Online Resource 4e). Increased activation of caspase-3 was similarly observed in cells bearing inclusions of (GA)_50_-V5, (GA)_100_-V5 or (GA)_50_-HA (Online Resource 4f). Poly(GA)-induced cytotoxicity was further confirmed by increased LDH levels in culture media of GFP-(GA)_50_-expressing cells compared to LDH levels in media from cells expressing either GFP alone or GFP-(GA)_5_ (Fig. 2h), despite comparable levels of transgene mRNA (Fig. 2i).

### Expression of poly(GA) proteins causes ER stress and neuronal death

**Fig. 3:**
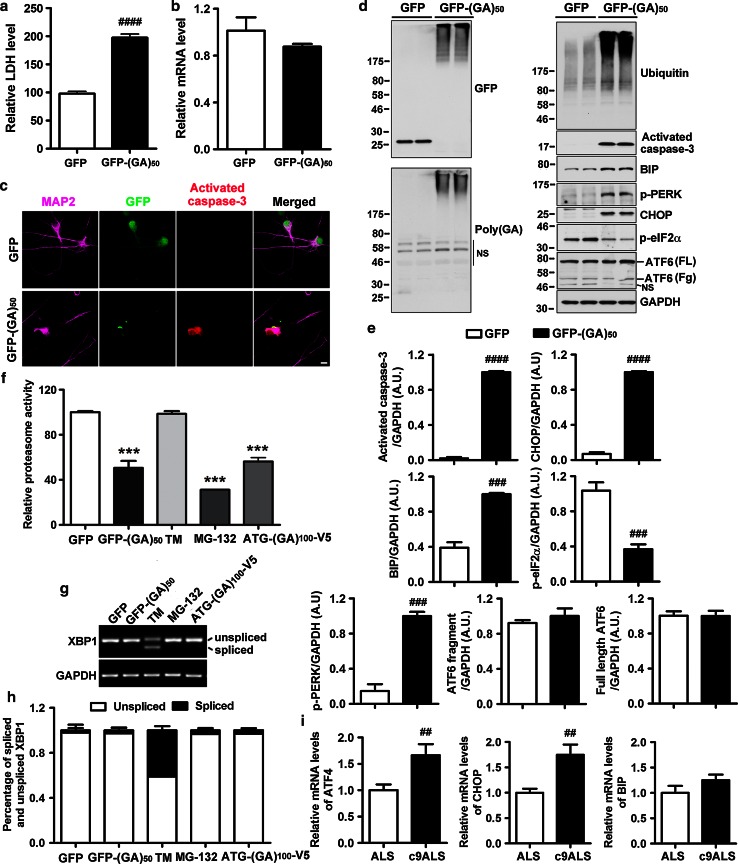
Expression of poly(GA) proteins in primary neurons causes neurotoxicity accompanied by UPS impairment and ER stress. **a** Expression of GFP-(GA)_50_, but not GFP, causes toxicity, as assessed by measuring LDH activity in media. **b** Transgene mRNA levels are comparable in neurons expressing GFP and GFP-(GA)_50_. **c** Activated caspase-3 is observed in MAP2-positive neurons bearing GFP-(GA)_50_ inclusions. *Scale bar* represents 10 μm. Western blot **(d)** and densitometric analysis of blots (**e**) show that expression of GFP-(GA)_50_ in primary neurons leads to increased levels of activated caspase-3 and ubiquitinated proteins. In addition, levels of the ER stress markers, BIP, phospho-PERK and CHOP, are increased by GFP-(GA)_50_ expression, whereas levels of phospho-eIF2α are decreased and ATF6 levels remain unchanged. *FL*, *Fg* bands corresponding to full-length and fragmented ATF6, respectively. *NS* non-specific bands. **f** Proteasome activity assays reveal that expression of poly(GA) proteins inhibit proteasome activity. Decreased proteasome activity is also observed in neurons treated with the proteasome inhibitor MG-132, but not with the ER stress inducer, tunicamycin. RT-PCR (**g**) and quantitative analysis (**h**) show that tunicamycin induces abnormal splicing of XBP1. However, expression of poly(GA) proteins or treatment with MG-132 does not result in this alteration. **i** mRNA levels of ER stress markers, ATF4 and CHOP, are significantly increased in frontal cortex of ALS patients with the *C9ORF72* repeat expansion. *N* = 8 for c9ALS and *N* = 11 for sporadic ALS without the *C9ORF72* repeat expansion. Data represents mean ± SEM of three separate experiments (**a**, **b**, **e**, **f**, **h**). ^##^ *P* < 0.01, ^###^ *P* < 0.001 and ^####^ *P* < 0.0001, as analyzed by unpaired t test. ***P < 0.001, as analyzed by one-way analysis of variance followed by Tukey’s post hoc analysis

Since GFP-(GA)_50_ expression in immortalized cultured cells caused a modest, but statistically significant, increase in caspase activation, we next sought to determine whether expression of poly(GA) proteins in primary neurons would result in inclusion formation and a similar or greater degree of toxicity. To this end, we generated adeno-associated viral vectors (AAV1) for the expression of GFP or GFP-(GA)_50_ for transduction of primary mouse cortical neurons at day in vitro 4. Seven days later, increased LDH levels were observed in medium from neurons expressing GFP-(GA)_50_ but not GFP (Fig. 3a), despite comparable levels of transgene mRNA (Fig. 3b). Additional signs of toxicity observed specifically in GFP-(GA)_50_-expressing neurons included caspase-3 activation in cells harboring inclusions (Fig. 3c–e) and stunted neurite outgrowth (Online Resource 5a and b). Impaired neurite outgrowth was also observed in primary neurons expressing ATG-(GA)_100_-V5 (Online Resource 5a and b), the expression of which resulted in the formation of poly(GA) inclusions positive for ubiquitin and p62 (Online Resource 5c).

Similarly to our findings above, immuno-EM studies showed that poly(GA) inclusions in GFP-(GA)_50_-expressing neurons were composed of filaments (arrow, Online Resource 5d), and that the expression of GFP-(GA)_50_, but not GFP alone, resulted in the formation of high molecular weight species (Fig. 3d). GFP-(GA)_50_ expression was also associated with the accumulation of ubiquitinated proteins (Fig. 3d), suggesting that poly(GA) proteins impair the activity of the ubiquitin–proteasome system (UPS). Indeed, proteasome activity was found to be significantly decreased in neurons expressing GFP-(GA)_50_ compared to those expressing GFP (Fig. 3f).

Since it is well established that proteasome inhibition causes ER stress [17, 32, 47, 50, 53], our findings may indicate that expression of poly(GA) proteins leads to ER stress. Although poly(GA) inclusions did not localize to the ER-Golgi secretory compartment (Online Resource 5e), poly(GA) expression nonetheless increased levels of binding immunoglobulin protein (BIP), an ER chaperone protein, phosphorylated protein kinase RNA (PKR)-like ER kinase (PERK), a critical transducer of ER stress, and transcriptional factor C/EBP homologous protein (CHOP), a downstream target of PERK (Fig. 3d, e), indicative of activation of the PERK–CHOP ER stress-associated signaling pathway. However, poly(GA) expression did not influence other pathways normally triggered by ER stress; neither induction of the activating transcription factor 6 (ATF6) axis, assessed by ATF6 cleavage (Fig 3d, e), nor of the inositol-requiring protein-1 (IRE1) axis, assessed by abnormal X-box-binding protein 1 (XBP1) splicing (Fig. 3g, h), were observed in neurons expressing GFP-(GA)_50_. As a positive control, non-transduced neurons were treated with tunicamycin, an ER stress inducer. This resulted in increased levels of BIP, PERK and CHOP (Online Resource 5f and g), as well as abnormal splicing of XBP1 (Fig. 3g, h), but not ATF6 cleavage (Online Resource 5f and g), suggesting that the ATF6 pathway is less likely to be activated upon ER stress in primary neurons. Of interest, tunicamycin treatment resulted in the accumulation of ubiquitinated proteins (Online Resource 5g) without direct inhibition of proteasome activity (Fig. 3f), suggesting increased ubiquitination of misfolded proteins in the ER.

Like GFP-(GA)_50_ expression, expression of ATG-(GA)_100_-V5 or exposure of neurons to the proteasome inhibitor, MG-132, resulted in the accumulation of ubiquitinated proteins, impairment of proteasome activity, as well as activation of caspase-3 and the PERK–CHOP pathway (Fig. 3f, Online Resource 5f and g). These findings provide further support that poly(GA) expression in primary neurons inhibits proteasome activity and leads to ER stress. We thus evaluated whether signs of heightened ER stress are observed in *C9ORF72* repeat expansion carriers. Given that TDP-43 pathology has been associated with ER stress [68, 70], and that both c9RAN protein pathology and TDP-43 pathology are present in c9ALS, we compared c9ALS cases to sporadic ALS cases with TDP-43 pathology to exclude the potentially confounding effect of TDP-43 on ER stress. We also chose to examine activating transcription factor 4 (ATF4) and CHOP, two ER stress markers involved in the PERK–CHOP pathway, given that poly(GA) protein expression in primary neurons specifically activates this pathway. Of importance, quantitative PCR analysis revealed that mRNA levels of ATF4 and CHOP were significantly increased in frontal cortex of c9ALS cases (Fig. 3i). BIP levels, however, did not differ (Fig. 3i).

### ER stress inhibitors, salubrinal and TUDCA, provide rescue against poly(GA)-induced ER stress and neurotoxicity

**Fig. 4:**
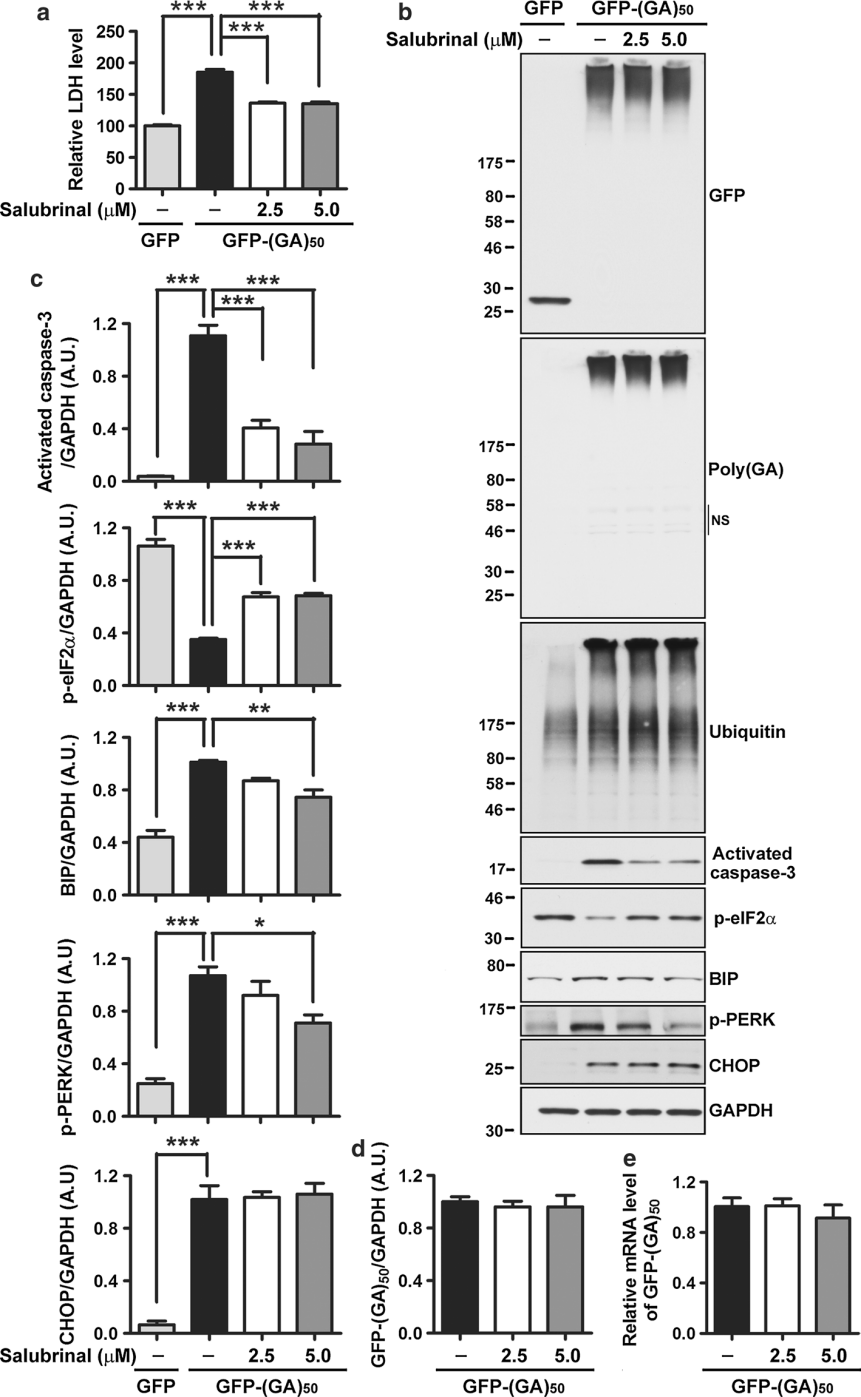
Salubrinal, a selective inhibitor of eIF2α dephosphorylation, protects neurons against poly(GA)-induced ER stress and toxicity. **a** Salubrinal, a small molecule known to provide rescue from ER stress and associated cell death by inhibiting the dephosphorylation of eIF2α, significantly decreases LDH activity in media of neurons expressing GFP-(GA)_50_. Western blot (**b**) and densitometric analysis of blots **(c)** show that treatment of GFP-(GA)_50_-expressing neurons with salubrinal significantly inhibits caspase-3 activation, increases eIF2α phosphorylation, and decreases levels of ER stress markers, BIP and phospho-PERK. Note that salubrinal treatment does not decrease levels of ubiquitinated proteins or CHOP. Levels of GFP-(GA)_50_ protein and mRNA are also not changed after salubrinal treatment, as shown by Western blot (**b**), densitometric analysis of blot (**d**), and qRT-PCR (**e**). *NS* indicates non-specific bands. Data represents mean ± SEM from three separate experiments. *P < 0.05, **P < 0.01 and ***P < 0.001, as analyzed by one-way analysis of variance followed by Tukey’s post hoc analysis

Since cell death associated with the PERK–CHOP signaling pathway is partially mediated through the dephosphorylation of eukaryotic translation initiation factor 2α (eIF2α), which is regulated by the association of growth arrest and DNA damage inducible 34 (GADD34) with protein phosphatase 1 (PP1C) [24, 25, 42, 49], we examined the phosphorylation status of eIF2α in neurons expressing GFP-(GA)_50_. Western blot analysis revealed that, compared to GFP expression, expression of GFP-(GA)_50_ significantly reduced eIF2α phosphorylation (Figs. 3d, e, 4b, c). We thus evaluated the neuroprotective effects of salubrinal, a selective inhibitor of the GADD34–PP1C complex known to offer protection from ER stress-associated cell death [9, 36, 60], in neurons expressing GFP-(GA)_50_. Compared to vehicle-treated GFP-(GA)_50_-expressing neurons, LDH activity in media (Fig. 4a) and caspase-3 activation (Fig. 4b, c) were significantly decreased in neurons treated with salubrinal (2.5 and 5 µM). This was accompanied by a dramatic increase in eIF2α phosphorylation (Fig. 4b, c), indicating that salubrinal efficiently inhibits the activity of the GADD34–PP1C complex. Moreover, salubrinal at 5 µM decreased levels of BIP and phospho-PERK (Fig. 4b, c), although neither levels of GFP-(GA)_50_ mRNA and protein, ubiquitinated proteins, nor CHOP were altered (Fig. 4b–e).

**Fig. 5:**
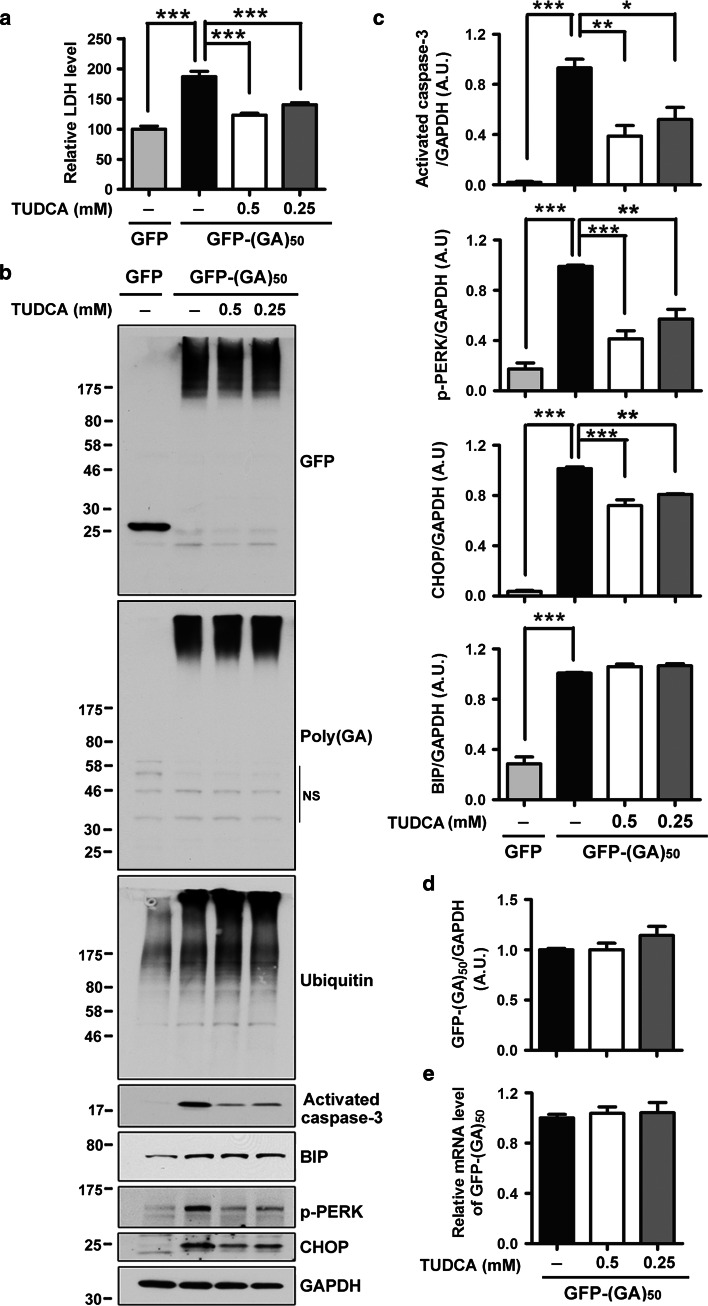
The chemical chaperone TUDCA protects neurons against poly(GA)-induced ER stress and toxicity. **a** TUDCA, a chemical chaperone known to inhibit ER stress and associated downstream pathways, significantly decreases LDH activity in media of neurons expressing GFP-(GA)_50_. **b**, **c** Treatment of GFP-(GA)_50_-expressing neurons with TUDCA also significantly inhibits caspase-3 activation, and decreases levels of ER stress markers, phospho-PERK and CHOP, as shown by Western blot (**b**) and densitometric analysis of blots (**c**). Note that TUDCA treatment does not decrease levels of ubiquitinated proteins and BIP (**b**, **c**). Protein and mRNA levels of GFP-(GA)_50_ are not changed after TUDCA treatment (**d**, **e**). *NS* non-specific bands. Data represents mean ± SEM from three separate experiments. *P < 0.05, **P < 0.01 and ***P < 0.001, as analyzed by one-way analysis of variance followed by Tukey’s post hoc analysis

To further evaluate the role of ER stress in poly(GA)-induced toxicity, neurons expressing GFP-(GA)_50_ were treated with the chemical chaperone TUDCA, an inhibitor of ER stress and associated toxicity [13, 38, 52, 72]. Compared to vehicle-treated neurons expressing GFP-(GA)_50_, those treated with TUDCA (0.25 and 0.5 mM) showed a significant decrease in LDH activity in media (Fig. 5a) and caspase-3 activation (Fig. 5b, c). In addition, a dramatic decrease in phospho-PERK and CHOP levels were observed (Fig. 5b, c). Conversely, levels of GFP-(GA)_50_ mRNA and protein, ubiquitinated proteins, and BIP were not altered by TUDCA treatment (Fig. 5b–e). Taken together, these findings indicate that inhibitors of ER stress provide protection against poly(GA) neurotoxicity.

## Discussion

RAN translation is becoming a well-established phenomenon in many repeat expansion disorders [3, 44, 65, 77]. Inclusions composed of RAN-translated proteins are now considered a neuropathological hallmark of c9FTD/ALS [3, 20, 43, 44, 78], but the contribution of this unconventional form of translation to disease pathogenesis has so far been enigmatic. While each c9RAN protein may influence neuronal health differently, herein we provide evidence that poly(GA) proteins are neurotoxic and could thus be implicated in the neurodegenerative processes of c9FTD/ALS.

Using cultured cells as a model to express c9RAN proteins of 50 repeating dipeptides, we observed that poly(GA) proteins formed ubiquitin- and p62-positive cytoplasmic inclusions, recapitulating the poly(GA) pathology of c9FTD/ALS [2, 44]. In contrast, GFP-(PA)_50_ and GFP-(GP)_47_ remained diffusely distributed, whereas GFP-(GR)_50_ and GFP-(PR)_50_ aggregated in the nucleus, perhaps as a result of repeat length and the fact that arginine (R)-rich regions can function as a nuclear localization signal. Because most poly(GR) and poly(PR) inclusions in c9FTD/ALS patients are cytoplasmic [20, 43, 44, 78], the exclusively nuclear localization of GFP-(GR)_50_ and GFP-(PR)_50_ inclusions in cultured cells does not accurately reflect c9FTD/ALS neuropathology. These studies indicate that poly(GA) proteins are highly aggregation-prone compared to the other c9RAN proteins, and that, depending on the model, longer repeat lengths should be used to evaluate poly(GP), poly(GR), poly(PA), and poly(PR) aggregation and toxicity to better mimic c9FTD/ALS.

To study the neuropathologic profile of poly(GA) inclusions in c9FTD/ALS, we generated a novel anti-poly(GA) antibody using as the antigen-purified recombinant GST-(GA)_50_, which formed high molecular weight material and filaments in vitro. Consistent with previous reports [40, 44, 56], we found that poly(GA) proteins were highly insoluble in c9FTD/ALS brain tissue, and that anti-poly(GA) immunoreactive inclusions were abundant throughout the CNS. To our knowledge, we are the first to report that poly(GA) proteins form filamentous structures in c9FTD/ALS brain tissue. Similar filamentous structures were produced in experimental models; we show that poly(GA) proteins with as little as 50 dipeptide-repeats self-assembled and formed filaments in vitro, as well as in cultured cells and primary neurons. It was with some surprise that we found, using fluorescent microscopy of live cells, that poly(GA) proteins quickly (within mere minutes) converted from a diffuse distribution to compact, small aggregates. Perhaps this rapid transformation occurs when the concentration of poly(GA) proteins within a localized area meets a critical threshold. These small aggregates may then serve to seed the aggregation of soluble poly(GA) proteins, thus causing larger inclusions to form. Poly(GA) may similarly seed the aggregation of other proteins, including c9RAN proteins, given that poly(GA) proteins have been shown to co-localize with various c9RAN proteins in neuronal inclusions in c9FTD/ALS [43, 44]. Like poly(GA) proteins, polyA proteins form high molecular weight species and inclusions in spinocerebellar ataxia type 8 (SCA8) [77], further indicating that proteins with long stretches of hydrophobic residues are especially aggregation-prone.

Cultured cell and primary neuron models provide evidence that expression and aggregation of poly(GA) proteins cause cellular toxicity. Compared to cells expressing GFP-(GA)_5_, which remained diffusely localized, there was enhanced cytotoxicity in cells expressing GFP-(GA)_50_, which formed cytoplasmic inclusions. The majority of GFP-(GA)_50_-expressing cells immunopositive for active caspase-3 contained such inclusions, suggesting that poly(GA) aggregation drives cell death. Nonetheless, it remains possible that soluble and insoluble poly(GA) oligomers also play a toxic role, as is speculated to be the case of inclusion-forming proteins, such as tau and TDP-43, in other neurodegenerative diseases. Whatever the neurotoxic poly(GA) species may be, our studies demonstrate that this c9RAN protein impairs function of the UPS in primary neurons. Compared to neurons expressing GFP alone, proteasome activity was significantly decreased in neurons expressing GFP-(GA)_50_, and this was accompanied with a dramatic accumulation of ubiquitinated proteins. These findings are consistent with reports demonstrating that protein aggregates and/or oligomers can impair the UPS [6, 7, 30, 35, 71].

ER-associated protein degradation (ERAD), which controls protein homeostasis in the ER, is dependent on proper UPS activity [10, 12]; consequently, inhibition of the UPS impairs ERAD, leading to the accumulation of misfolded proteins in the ER and ER stress [17, 32, 47, 50, 53, 74]. Under conditions of ER stress, BIP, a master regulator of the unfolded protein response (UPR) that normally binds to and inhibits activity of ER stress sensors, PERK, ATF6 and IRE1, dissociates from these proteins. This disassociation leads to the activation of different downstream signaling pathways such as PERK auto-phosphorylation, ATF6 cleavage and XBP-1 splicing [25, 67]. Of note, the PERK–CHOP pathway was selectively activated in primary neurons expressing GFP-(GA)_50_, as evidenced by increased levels of the ER sensor phospho-PERK, and its downstream target CHOP. Selective activation of the PERK–CHOP pathway was similarly observed in non-transduced neurons treated with the proteasome inhibitor, MG-132. Other similarities between GFP-(GA)_50_ expression and MG-132-induced proteasome inhibition include the accumulation of ubiquitinated proteins, decreased proteasome activity, and caspase-3 activation. In contrast, treatment of neurons with tunicamycin, an ER stress inducer, activated both PERK–CHOP and IRE–XBP1 pathways without impairing proteasome activity. Together, our findings suggest that poly(GA) proteins indirectly induce ER stress through proteasome inhibition. Of importance, we show that mRNA levels of ER stress markers of the PERK–CHOP pathway, ATF4 and CHOP, are significantly increased in c9ALS cases compared to sporadic ALS cases without the *C9ORF72* repeat expansion. While no difference in BIP mRNA levels were observed, this may be due to the shared TDP-43 pathology between *C9ORF72*-positive and -negative ALS, which has been associated with ER stress [68, 70]. In addition to our findings using human postmortem samples, it is also worth noting that a recent study reported that iPSC-derived motor neurons from c9ALS patient, which contain RNA foci and c9RAN proteins but no TDP-43 pathology [15], exhibit an increased vulnerability to tunicamycin [23], supporting the association between the *C9ORF72* repeat expansion and ER stress. Future studies investigating whether, like poly(GA) proteins, RNA foci and other c9RAN proteins inhibit proteasome activity and cause ER stress are warranted.

ER stress activation of the PERK–CHOP pathway is well documented (see [25, 67] for review). In brief, PERK is activated through auto-phosphorylation upon release from BIP. Upon activation, PERK phosphorylates eIF2α to attenuate global protein synthesis as a pro-survival mechanism. However, phosphorylation of eIF2α selectively allows the translation of ATF4 and CHOP, which induce cell death under prolonged ER stress. This can occur through multiple mechanisms that include expression of pro-apoptotic genes, promotion of protein synthesis, production of reactive oxygen species (ROS) and ATP depletion [18, 24, 25, 42, 51, 63]. For example, ATF4 and CHOP up-regulate levels of GADD34, which forms a complex with PP1C to dephosphorylate eIF2α; the dephosphorylation of eIF2α in turn enhances protein synthesis, ATP depletion and ROS production, which consequently induce cell death [24, 42]. The importance of this pathway is further validated by studies showing selective inhibitors of the GADD34–PP1C complex protect cells from ER stress-induced cell death [9, 66]. Consistent with these findings, we show that salubrinal, a selective inhibitor of the GADD34–PP1C complex [9, 36, 60], restored eIF2α phosphorylation, reduced ER stress and increased cell survival in neurons expressing poly(GA) proteins. In addition, TUDCA, an inhibitor of ER stress [13, 38, 52, 72], blocked the increase in phospho-PERK and CHOP normally observed in poly(GA)-expressing neurons, and provided neuroprotection against poly(GA)-induced toxicity. Of interest, the levels of poly(GA) and ubiquitinated proteins were not themselves decreased after salubrinal and TUDCA treatment, suggesting that proteasome inhibition, the culprit responsible for inducing ER stress, remained present in these cells. This may explain why levels of BIP, which correlate with the amount of misfolded proteins in the ER, were not altered following treatment of GFP-(GA)_50_-expressing cells with TUDCA. As a chemical chaperone, TUDCA may have bound to these misfolded proteins and caused the release of BIP, which then became free to interact with PERK and inhibit PERK auto-phosphorylation and activation. As a result, CHOP levels decreased as did caspase-3 activation. However, BIP levels remain unchanged because TUDCA did not eliminate the accumulation of misfolded proteins, but rather targeted downstream events only. Conversely, salubrinal treatment would have decreased protein translation by enhancing eIF2α phosphorylation, thus reducing the misfolded protein load in the ER. Consequently, this would cause a decrease in levels of BIP and phospho-PERK, and increase cell survival. That CHOP levels were not also decreased by salubrinal treatment may result from the increase in eIF2α phosphorylation, which regulates CHOP expression.

In summary, we have generated a novel poly(GA) antibody and confirmed that abundant poly(GA) neuronal inclusions are detected throughout the CNS of c9FTD/ALS cases. We show that poly(GA) proteins are highly aggregation-prone and form filamentous structures in experimental models and c9FTD/ALS brain tissue. Of particular importance, our data provide compelling evidence that poly(GA) proteins contribute to the neurodegeneration in c9FTD/ALS. The expression of poly(GA) proteins causes neurotoxicity; this toxicity occurs in the absence of RNA foci, and is associated with impairment of the UPS and induction of ER stress. ER stress is believed to play an important role in several neurodegenerative diseases, including sporadic ALS [4, 26, 59, 69], and familiar ALS caused by mutations in Cu/Zn superoxide dismutase [8, 29, 48, 55] or vesicle-associated membrane protein-associated protein B [27, 45, 62]. Our data extend the list of diseases involving ER stress, and suggest that targeting the ER, using small molecules such as salubrinal and TUDCA, may be a promising therapeutic approach for c9FTD/ALS.

## Electronic supplementary material

Below is the link to the electronic supplementary material.

Supplementary material 1 (TIFF 1510 kb)

Supplementary material 2 (TIFF 1012 kb)

Supplementary material 3 (MPG 596 kb)

Supplementary material 4 (TIFF 3146 kb)

Supplementary material 5 (TIFF 2658 kb)

Supplementary material 6 (DOCX 4962 kb)
