# Supplementary figures and images for: Aggregation-prone c9FTD/ALS poly(GA) RAN-translated proteins cause neurotoxicity by inducing ER stress
Source: Acta Neuropathol. 2014 Aug 31;128(4):505–24. doi: 10.1007/s00401-014-1336-5 (PMC4159567; doi:10.1007/s00401-014-1336-5)

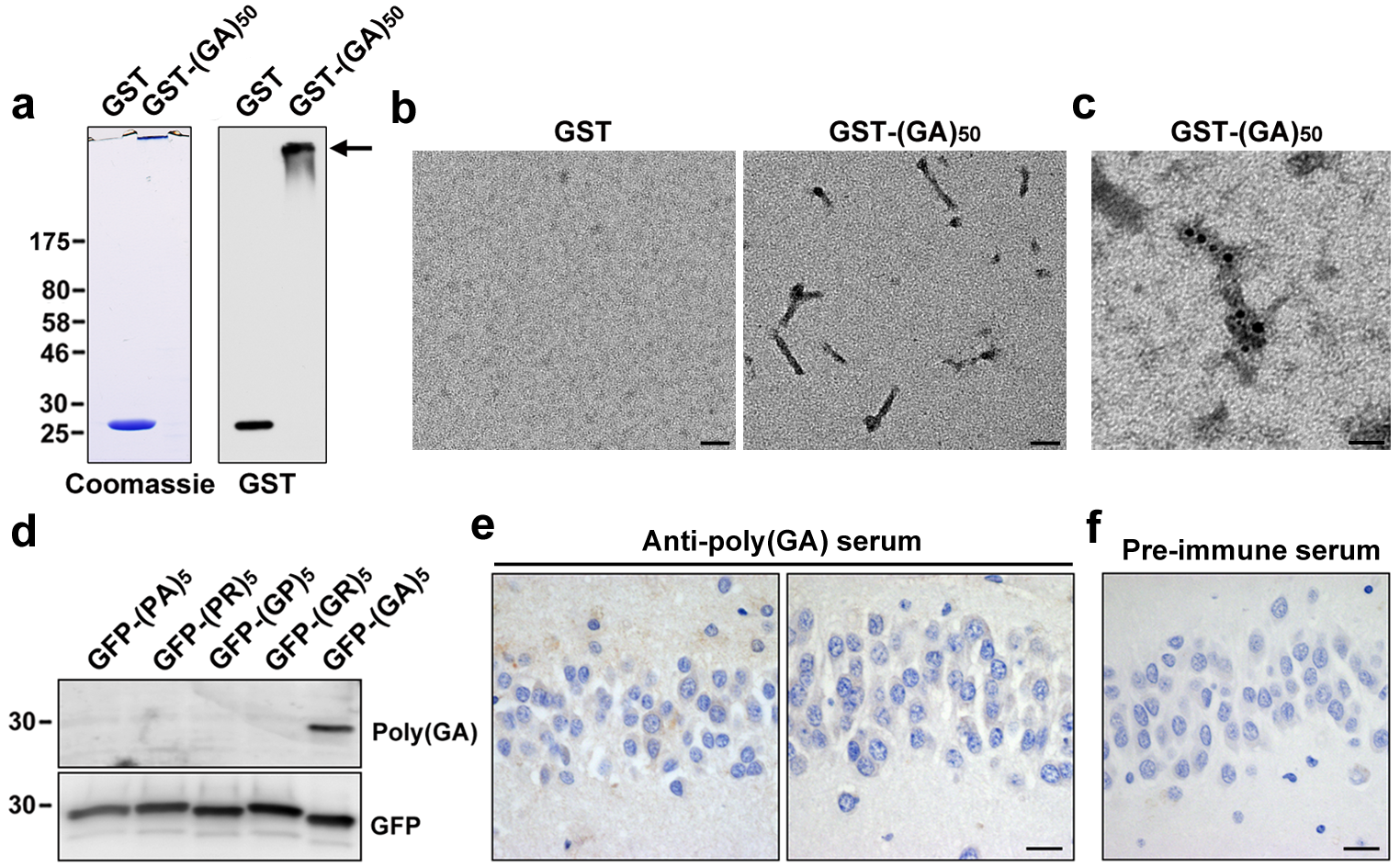

Supplement: Supplementary file 1 — Supplementary material 1 (TIFF 1510 kb) [file 401_2014_1336_MOESM1_ESM.tif]

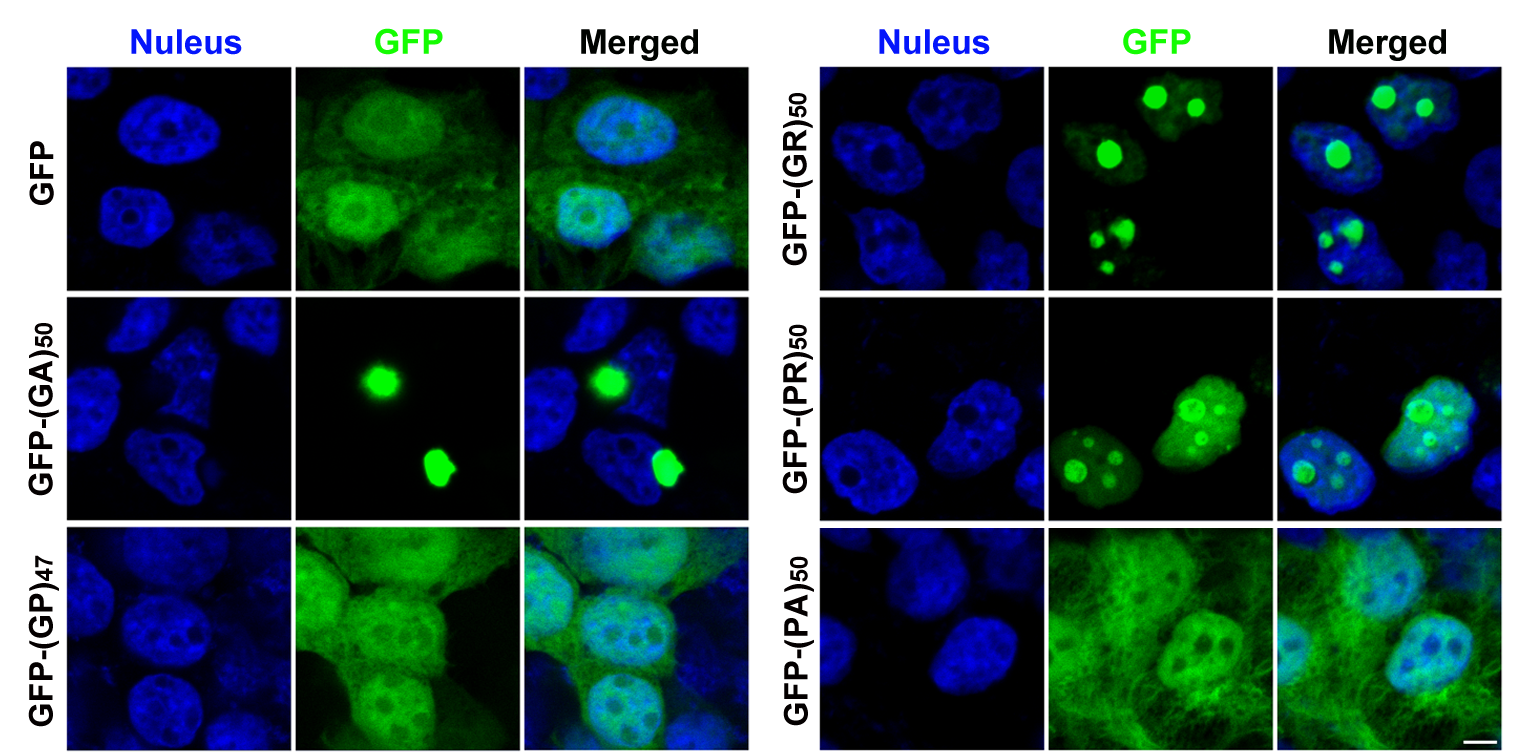

Supplement: Supplementary file 2 — Supplementary material 2 (TIFF 1012 kb) [file 401_2014_1336_MOESM2_ESM.tif]

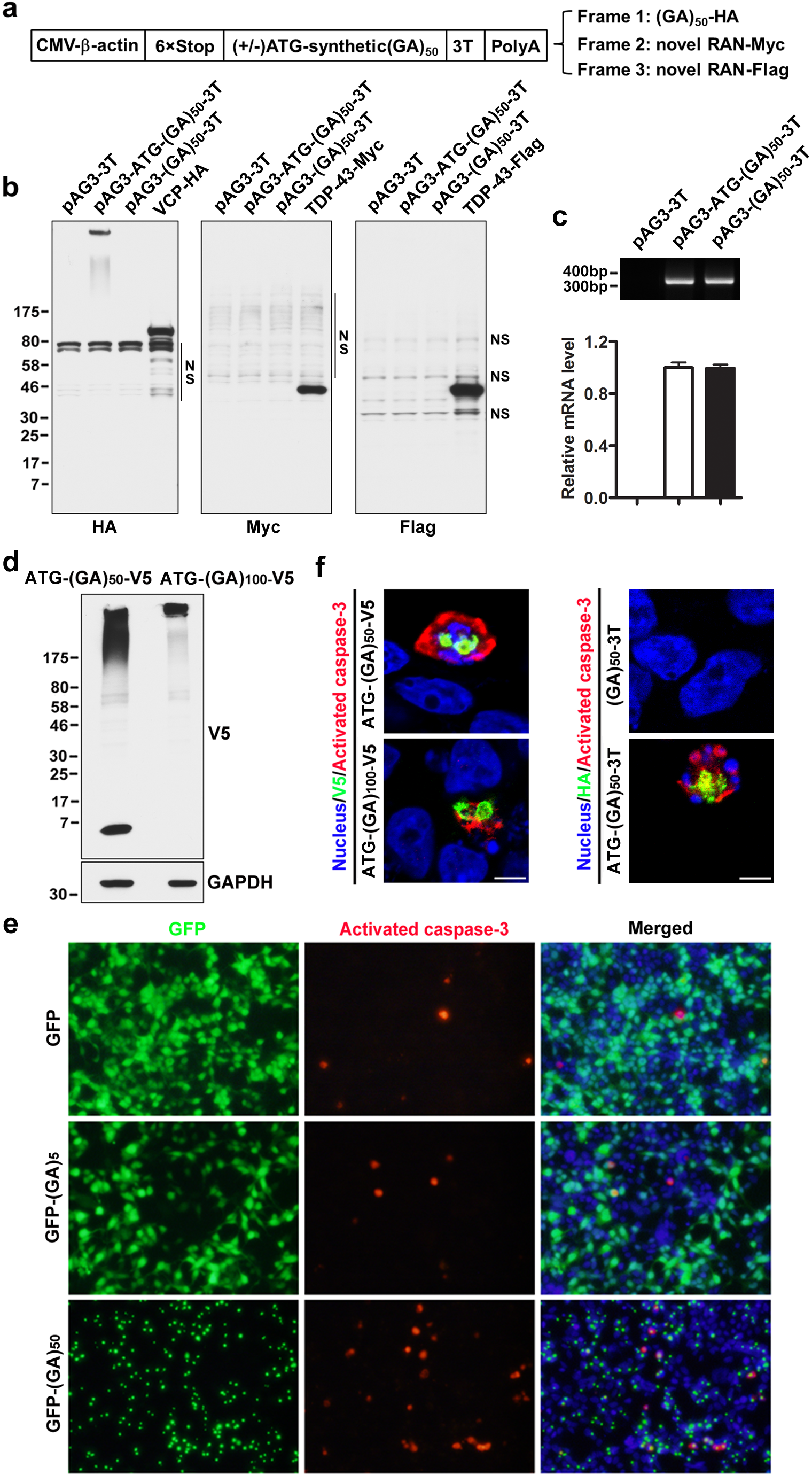

Supplement: Supplementary file 4 — Supplementary material 4 (TIFF 3146 kb) [file 401_2014_1336_MOESM4_ESM.tif]

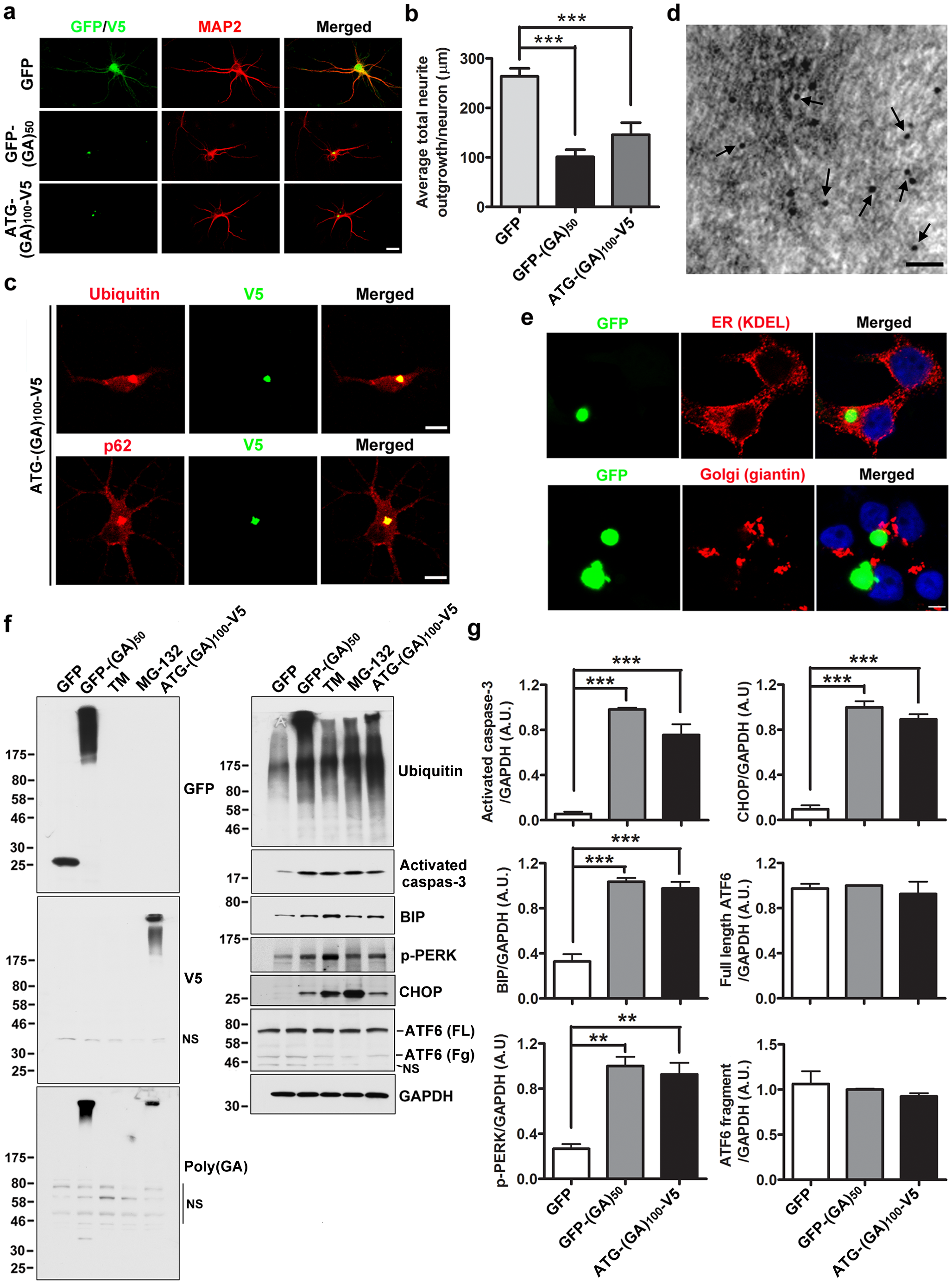

Supplement: Supplementary file 5 — Supplementary material 5 (TIFF 2658 kb) [file 401_2014_1336_MOESM5_ESM.tif]
