# Supplementary material for: Aggregation-prone c9FTD/ALS poly(GA) RAN-translated proteins cause neurotoxicity by inducing ER stress
Source: Acta Neuropathol. 2014 Aug 31;128(4):505–24. doi: 10.1007/s00401-014-1336-5 (PMC4159567; doi:10.1007/s00401-014-1336-5)
Supplement: Supplementary file 6 — Supplementary material 6 (DOCX 4962 kb) [file 401_2014_1336_MOESM6_ESM.docx]

**Acta Neuropathologica**

**Electronic Supplementary Material**

**Aggregation-prone c9FTD/ALS poly(GA) RAN translated proteins cause neurotoxicity by inducing ER stress**

**Yong-Jie Zhang^1^, Karen Jansen-West^1^, Ya-Fei Xu^1^, Tania F. Gendron^1^, Kevin F. Bieniek^1,^**^2^**, Wen-Lang Lin^1^, Hiroki Sasaguri^1^, Thomas Caulfield^1^, Jaime Hubbard^1^, Lillian Daughrity^1^, Jeannie Chew^1,^**^2^**, Veronique V. Belzil^1^, Mercedes Prudencio^1^, Jeannette N. Stankowski^1^, Monica Castanedes-Casey^1^, Ena Whitelaw^1^, Peter E. A. Ash**^3^**, Michael DeTure^1^, Rosa Rademakers^1^, Kevin B. Boylan^4^, Dennis W. Dickson^1^, Leonard Petrucelli^1^***

^1^ Department of Neuroscience, Mayo Clinic Florida, Jacksonville, FL 32224, USA.

^2^ Mayo Graduate School, Mayo Clinic College of Medicine, Rochester, MN 55905, USA.

^3^ Department of Pharmacology, Boston University School of Medicine, Boston, MA 02118, USA.

^4^ Department of Neurology, Mayo Clinic Florida, Jacksonville, FL 32224, USA

* Corresponding author:

Leonard Petrucelli

Tel: 904-953-2855

Email: [Petrucelli.Leonard@mayo.edu](mailto:Petrucelli.Leonard@mayo.edu)

**
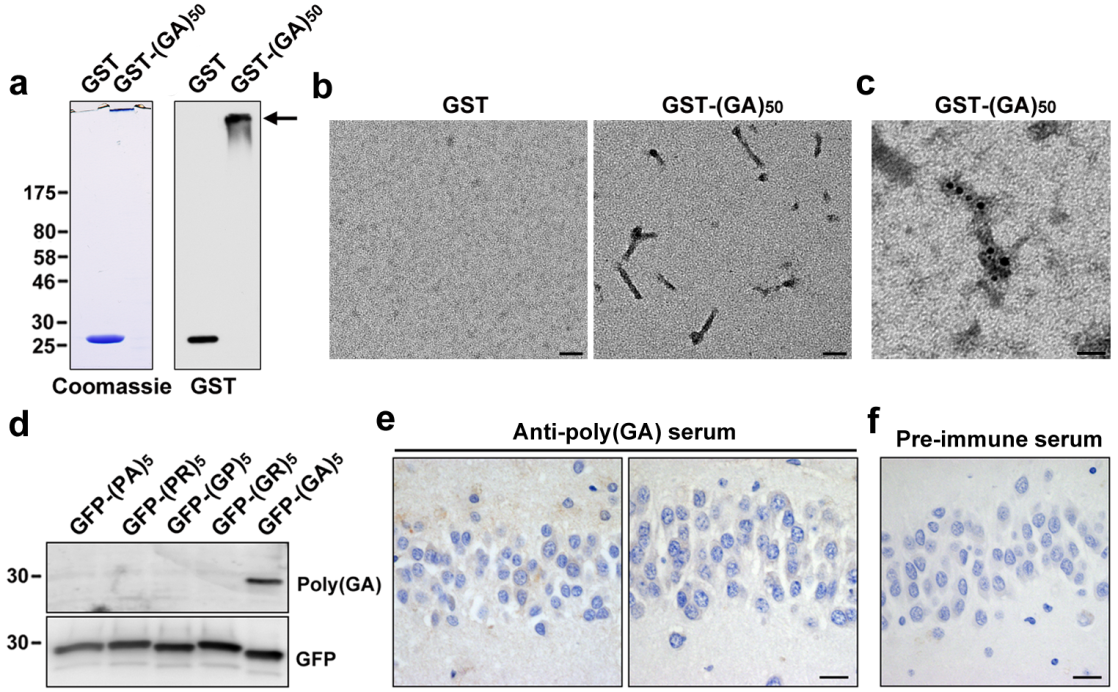
**

**Online Resource 1. Generation and characterization of anti-poly(GA) antibody. (a)** Coomassie blue staining (left) and Western blot analysis (right) show that recombinant GST-(GA)_50_ proteins form high molecular weight species (*arrow*). In contrast, recombinant GST alone is detected as a monomer. **(b)** Electron microscopy (EM) analysis reveals that recombinant GST-(GA)_50_ proteins (right), but not GST alone (left), assemble into filaments with a regular structural morphology. *Scale bar* represents 50 nm. **(c)** Immuno-EM with anti-GST antibody labeled with gold particles (6 nm) confirm the localization of poly(GA) proteins to the filaments. *Scale bar* represents 20 nm. **(d)** Western blot analysis of lysates from HEK293T cells transfected to express GFP-tagged (PA)_5_, (PR)_5,_ (GP)_5_, (GR)_5_, (GA)_5_ or (GA)_50_, which represent all c9RAN proteins, confirms that anti-poly(GA) antibody specifically detects poly(GA) proteins. NS indicates non-specific bands. **(e)** No poly(GA) pathology is detected in *C9ORF72*-negative hippocampal dentate fascia immunostained with anti-poly(GA) antibody. **(f)** No immunoreactivity is observed in c9FTD/ALS hippocampal dentate fascia stained with pre-immune serum. *Scale bars* in (**e**) and (**f**) represent 20 μm.

**
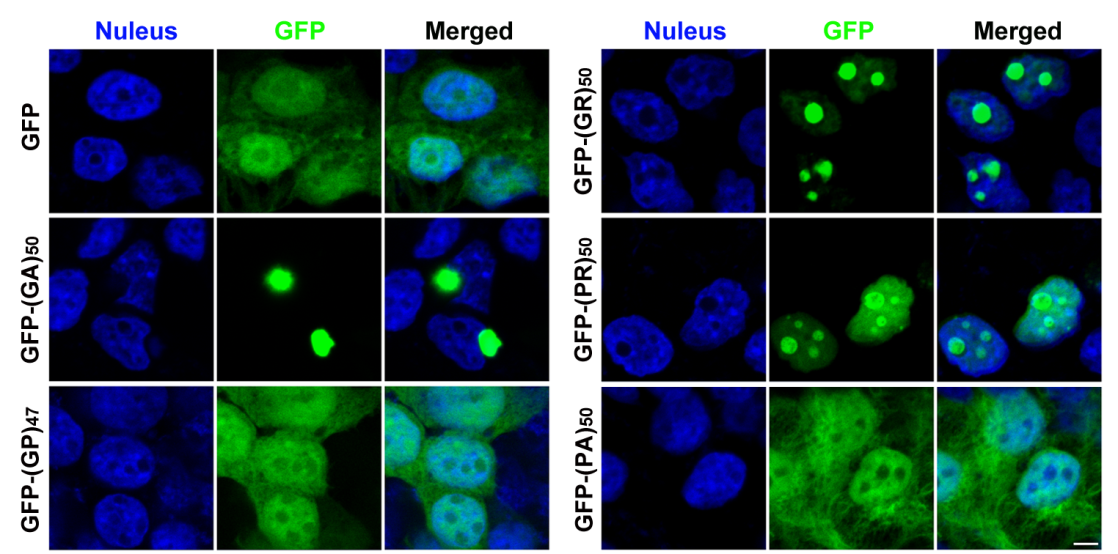
**

**Online Resource 2. Comparison of cellular distribution and aggregation properties among c9RAN proteins of 50 dipeptide repeats in cultured cells.** Confocal microscopy analysis of HEK293T cells expressing GFP-tagged c9RAN proteins reveals that, similar to GFP alone, GFP-(GP)_47_ and GFP-(PA)_50_ are diffusely distributed throughout cells. In contrast, GFP-(GA)_50_ forms cytoplasmic inclusions, whereas GFP-(GR)_50_ and GFP-(PR)_50_ accumulate into discrete nuclear structures. *Scale bar* represents 5 µm.

**Online Resource 3. Live cell imaging to monitor inclusion formation in cultured cells expressing GFP-(GA)_50_.** To monitor inclusion formation, images of live cells expressing GFP-(GA)_50_ were taken every 5 min for 5 hours using the BD pathway 855. The resulting images were combined in order to create a video.


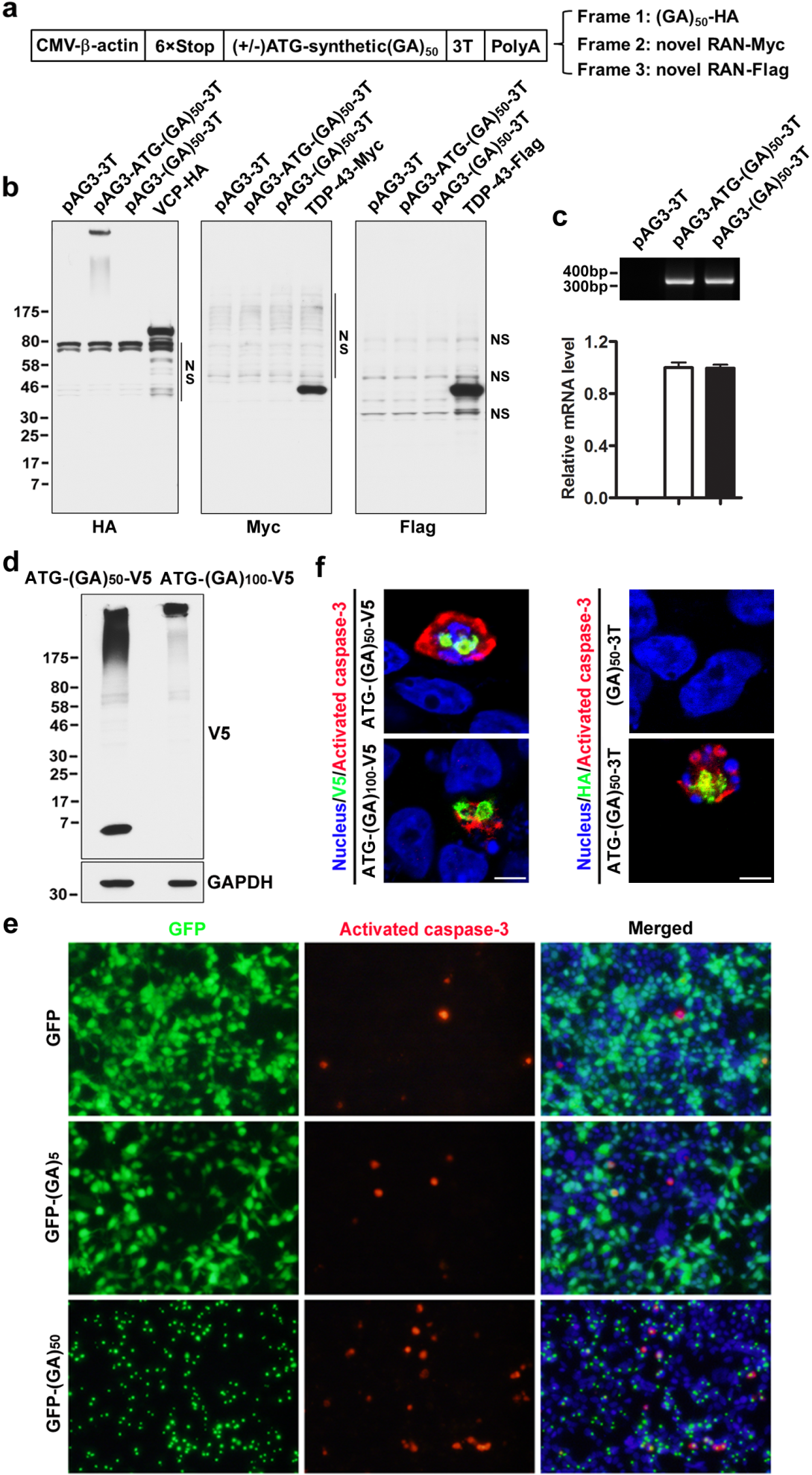


**Online Resource 4. Poly(GA) proteins form inclusions and induce toxicity in cultured cells.** **(a)** Schematic representation of ATG-(GA)_50_-3T and (GA)_50_-3T expression vectors, which include six stop codons upstream of (+/-)ATG-(GA)_50_ and a tag in each reading frame downstream of (GA)_50_ [i.e., frame 1: (GA)_50_-HA; frame 2: novel RAN protein-Myc; frame 3: novel RAN protein-Flag]. **(b)** Western blot analysis revealed that expression of ATG-(GA)_50_-3T in cells results in the production of (GA)_50_-HA proteins but not of Myc- or Flag-tagged proteins RAN translated from the second or third reading frames. Of note, (GA)_50_-HA proteins form high molecular weight material. No proteins are RAN translated from any of the three reading frames in cells transfected with the vector for non-ATG initiated (GA)_50_-3T expression despite exposing blots long enough to detect non-specific (NS) bands. VCP-HA, TDP-43-Myc and TDP-43-Flag were used as controls to ensure proper detection of tags by the antibodies employed. (**c**) mRNA levels between ATG-(GA)_50_-3T and (GA)_50_-3T are comparable. Data represents mean ± SEM of three separate experiments. (**d**) Western blot analysis shows that high molecular weight poly(GA) proteins are observed in cells expressing ATG-(GA)_50_-V5 or ATG-(GA)_100_-V5. **(e)** Representative images showing GFP- or GFP-(GA)_n_-expressing cells (green) immunostained for activated caspase-3 (red). Note that cultures expressing GFP-(GA)_50_ show increased activated caspase-3 staining, especially in inclusion-bearing cells, in comparison to cultures expressing GFP or GFP-(GA)_5_. 20× magnification. **(f)** Immunofluorescence staining reveals that expression of ATG-(GA)_50_-V5, ATG-(GA)_100_-V5 or ATG-(GA)_50_-HA also results in inclusion formation (green) and caspase-3 activation (red). Consistent with Western blot analysis (panel **b**), no poly(GA) proteins are expressed in cells transfected with the vector for non-ATG initiated (GA)_50_-3T expression. *Scale bar* represents 5 µm.

**
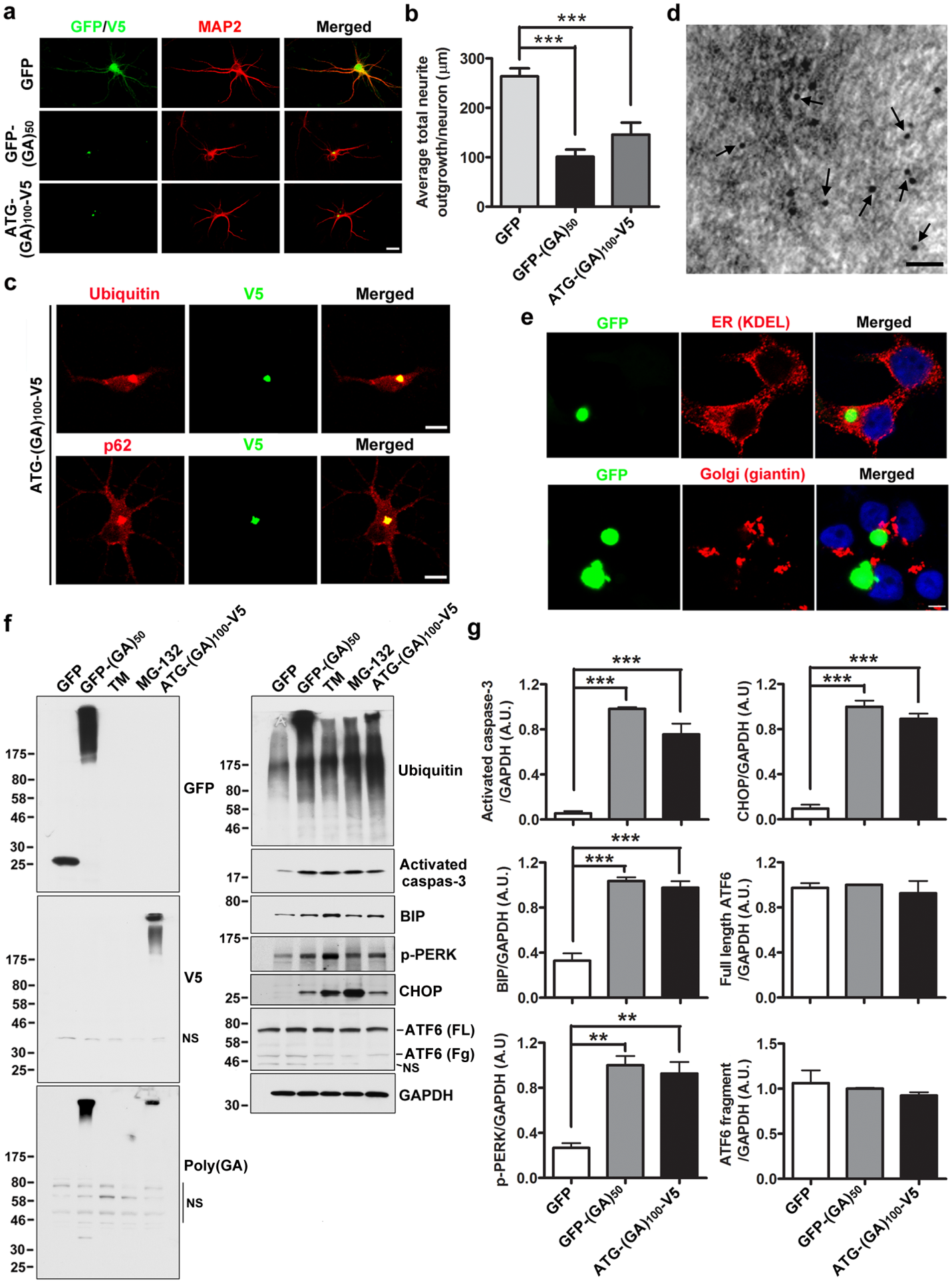
**

**Online Resource 5. Expression of poly(GA) proteins in primary neurons results in neurotoxicity accompanied by UPS impairment and induction of ER stress.** Representative images **(a)** and quantitative analysis **(b)** reveal that, compared with the expression of GFP alone, the expression of GFP-(GA)_50_ or ATG-(GA)_100_-V5 in primary cortical neurons significantly impairs neurite outgrowth, as indicated by a decrease in the average total length of microtubule-associated protein 2 (MAP2)-positive neurites. *Scale bar* represents 20 µm. (**c**) Poly(GA) inclusions in neurons expressing ATG-(GA)_100_-V5 are positive for ubiquitin and p62. *Scale bar* represents 10 µm. **(d)** Immuno-electron microscopy analysis of GFP-(GA)_50_-expressing neurons stained with an anti-GFP antibody labeled with gold particles confirms the localization of poly(GA) proteins to filaments in cytoplasmic inclusions (*arrow*). *Scale bar* represents 100 nm. **(e)** GFP-(GA)_50_ inclusions do not colocalize with the ER marker, KDEL, or the Golgi marker, giantin. *Scale bar* represents 5 µm. Western blot analysis **(f)** and densitometric analysis of blots (**g**) shows that, similar to GFP-(GA)_50_, expression of ATG-(GA)_100_-V5 activates caspase-3, causes the accumulation of ubiquitinated proteins, and increases BIP, phospho-PERK and CHOP levels indicative of ER stress. Compared with poly(GA) levels in GFP-(GA)_50_-expressing neurons, levels of poly(GA) protein are lower in neurons expressing ATG-(GA)_100_-V5. Nonetheless, the degree of ER stress induction appears comparable between both, suggesting that poly(GA) proteins induce similar effects when their levels reach a certain threshold. Note that proteasome inhibition by MG-132 and induction of ER stress by tunicamycin cause changes to occur in primary neurons similar to those observed in neurons expressing poly(GA) proteins. FL and Fg represent full-length and fragmented ATF6, respectively. NS indicates non-specific bands. Data represents mean ± SEM of three separate experiments. ***P<0.01, as assessed by one-way analysis of variance followed by Tukey’s post-hoc analysis.
